# Nitrogen-Rich Porous Organic Polymers with Supported Ag Nanoparticles for Efficient CO_2_ Conversion

**DOI:** 10.3390/nano12183088

**Published:** 2022-09-06

**Authors:** Jinyi Wu, Shasha Ma, Jiawei Cui, Zujin Yang, Jianyong Zhang

**Affiliations:** 1 MOE Laboratory of Polymeric Composite and Functional Materials, School of Materials Science and Engineering, Sun Yat-sen University, Guangzhou 510275, China; 2 School of Chemical Engineering and Technology, Sun Yat-sen University, Guangzhou 510275, China

**Keywords:** porous organic polymers, Ag nanoparticles, CO_2_ conversion, CO_2_ adsorption, multifunctional catalysis

## Abstract

As CO_2_ emissions increase and the global climate deteriorates, converting CO_2_ into valuable chemicals has become a topic of wide concern. The development of multifunctional catalysts for efficient CO_2_ conversion remains a major challenge. Herein, two porous organic polymers (NPOPs) functionalized with covalent triazine and triazole *N*-heterocycles are synthesized through the copper(I)-catalyzed azide–alkyne cycloaddition (CuAAC) reaction. The NPOPs have an abundant microporous content and high specific surface area, which confer them excellent CO_2_ affinities with a CO_2_ adsorption capacity of 84.0 mg g^−1^ and 63.7 mg g^−1^, respectively, at 273 K and 0.1 MPa. After wet impregnation and in situ reductions, Ag nanoparticles were supported in the NPOPs to obtain Ag@NPOPs with high dispersion and small particle size. The Ag@NPOPs were applied to high-value conversion reactions of CO_2_ with propargylic amines and terminal alkynes under mild reaction conditions. The carboxylative cyclization transformation of propargylic amine into 2-oxazolidinone and the carboxylation transformation of terminal alkynes into phenylpropiolic acid had the highest TOF values of 1125.1 and 90.9 h^−1^, respectively. The Ag@NPOP-1 was recycled and used five times without any significant decrease in catalytic activity, showing excellent catalytic stability and durability.

## 1. Introduction

The massive consumption of fossil energy has led to an increase in CO_2_ emissions in recent years, and the accompanying environmental problems are becoming progressively rigorous. Reducing CO_2_ emissions has been an imperative and urgent measure at the present moment [1,2]. To address this issue, the conversion of CO_2_ into high-value-added chemical products at atmospheric pressure is a promising approach, since CO_2_ is a sustainable and accessible C_1_ feedstock [3]. There have been reports on the conversion of CO_2_ into various valuable chemicals, including CO [4,5], CH_4_ [6,7], formic acid [8,9], methanol [10,11], cyclic carbonate [12,13], oxazolidinones [14,15], propargylic acid [16,17], etc. Considering the thermodynamic stability and kinetic inertia of CO_2_ [18], it is both challenging and groundbreaking to explore catalysts with efficient catalytic activity. Up to now, many catalytic systems have been applied to the high-value conversion of CO_2_, including zeolites [19,20], ionic liquids [21,22], inorganic salts [23,24], metal–organic frameworks (MOFs) [25,26], covalent organic frameworks (COFs) [27,28], porous organic polymers (POPs) [29,30], etc. Despite the previous efforts of many researchers, there are some deficiencies in the catalytic efficiency and catalytic scope of these systems; therefore, it is of great importance to develop catalysts with high efficiency and stability that can be applied to multiple scopes of high value-added conversions of CO_2_.

Covalent triazine frameworks (CTFs) are a novel catalogue of porous organic polymers. Due to their controllable functional framework, adjustable pore structure, high specific surface area, and excellent chemical stability, they have attracted extensive attention since Kuhn et al. reported the ionothermal synthesis in 2008 [31]. They have been widely used in various fields, including adsorption [32,33], separation [34,35], energy storage [36,37], and catalysis [38,39]. A large number of inherent pores and the rich content of nitrogen atoms in CTFs endow them with attractive affinity and adsorption capacity for CO_2_ [40,41]. Researchers have theoretically demonstrated the existence of van der Waals forces between the molecular dipole moment of CO_2_ and the negative electrostatic potential near the triazine-N atom [42]. Some reports show that CTFs can be applied to the conversion of CO_2_ [43,44]; however, due to a lack of metal active centers, the catalytic efficiency of the system still needs to be improved. The introduction of metal active centers into the CTF framework can greatly improve its catalytic efficiency for CO_2_ conversion [45]. As a powerful synthesis tool, the copper-catalyzed azide-alkyne cycloaddition (CuAAC) reaction has made rapid development and is widely employed in materials [46], biomedicine [47], and sensing [48] since its discovery by Sharpless in 2001 [49]. The triazole ring produced by the CuAAC reaction is rich in nitrogen atoms, and these nitrogen atoms have a strong coordination interaction with metals. A series of metal-supported porous organic polymers based on the CuAAC reaction have been reported to be applied in the catalysis field [50,51].

In this work, we construct two N-rich porous organic polymers (NPOPs) using the CuAAC reaction of 2,4,6-tris(4-ethynylphenyl)-1,3,5-triazine (TET) with tetrakis(4-azidophenyl)methane (TAM) and 1,4-diazidobenzene (DAB) based on the covalent triazine frameworks functionalized with triazole rings. Owing to the abundant nitrogen content in NPOPs, they show an excellent affinity and adsorption for CO_2_. Subsequently, the NPOPs are used as supports for Ag nanoparticles, and Ag@NPOPs are synthesized by wet impregnation and reduction. The Ag@NPOPs are catalytically active in the carboxycyclization of propargylamine and the carboxylation of terminal alkynes with CO_2_ to exhibit excellent catalytic activity and stability.

## 2. Materials and Methods

### 2.1. Synthesis of NPOP-1

The 2,4,6-Tris(4-ethynylphenyl)-1,3,5-triazine (157.5 mg, 0.41 mmol) and tetrakis(4-azidophenyl)methane (150.0 mg, 0.31 mmol) were dissolved in 120 mL DMF. Then Cu(PPh_3_)_3_Br (28.0 mg, 0.03 mmol) was added, and the resulting solution was heated at 100 °C for 48 h. The yellow solid was obtained by filtration and washed by acetone, CH_2_Cl_2_, and EtOH (30 mL) for three times in turn. Finally, the brownish-yellow solid was dried under reduced pressure at 60 °C for 6 h (270.6 mg, 88%).

### 2.2. Synthesis of NPOP-2

The 2,4,6-Tris(4-ethynylphenyl)-1,3,5-triazine (238.2 mg, 0.62 mmol) and 1,4-azido benzene (150.0 mg, 0.96 mmol) were dissolved in 120 mL DMF. Then Cu(PPh_3_)_3_Br (83.7 mg, 0.09 mmol) was added, and the resulting solution was heated at 100 °C for 48 h. The yellow solid was obtained by filtration and washed by acetone, CH_2_Cl_2_, and EtOH (30 mL) for three times in turn. Finally, the brownish-yellow solid was dried under reduced pressure at 60 °C for 6 h (326.1 mg, 84%).

### 2.3. Synthesis of Ag@NPOP-1

NPOP-1 (30.0 mg) was added to an AgNO_3_ aqueous solution (20 mmol L^−^^1^, 3.0 mL), and stirred for 12 h. Then the solid was washed by water (10 mL) three times. After that, the residue was added to 10 mL MeOH and stirred for 6 h. The reaction mixture was filtered and washed by water (10 mL) three times. Finally, the black solid was dried in a vacuum at 60 °C for 6 h.

### 2.4. Synthesis of Ag@NPOP-2

NPOP-2 (30.0 mg) was added to an AgNO_3_ aqueous solution (20 mmol L^−^^1^, 3.0 mL), and stirred for 12 h, then the solid was washed by water (10 mL) three times. After that, the residue was added to 10 mL MeOH and stirred for 6 h. The reaction mixture was filtered and washed by water (10 mL) three times. Finally, the black solid was dried in a vacuum at 60 °C for 6 h.

### 2.5. Carboxylative Cyclization of Propargylic Amines with CO_2_

In a typical experiment, propargylic amine (0.2 mmol), 1,8-diazabicyclo [5.4.0] undec-7-ene (DBU) (0.1 mmol), and the catalyst (1.0 mg) were mixed in acetonitrile (1.0 mL), and a CO_2_ balloon was equipped. CO_2_ was then charged into the reaction system after degassing and charging with CO_2_ three times. After the reaction mixture was stirred at 50 °C for 2 h, the catalyst was filtered and the yield was determined by ^1^H NMR with 1,4-dinitrobenzene as the internal standard. To obtain pure products, the concentrated crude product was purified by column chromatography.

### 2.6. Carboxylation of Terminal Alkyne

In a typical experiment, 1-ethynylbenzene (0.2 mmol) and the catalyst (1.0 mg) were mixed in DMSO (1.0 mL), and a CO_2_ balloon was equipped. CO_2_ was then charged into the reaction system after degassing and charging with CO_2_ three times. After the reaction mixture was stirred at 60 °C for 12 h, the system was cooled to room temperature. After the addition of water (2 mL), the solid was separated by filtration and washed with water (2 mL × 3). The filtrate was acidified with 1 mol L^−^^1^ HCl to pH = 1, and then extracted with CH_2_Cl_2_ (5 mL × 3). The yield of propiolic acid was determined by the ^1^H NMR. To obtain pure product, the concentrated crude product was purified by column chromatography.

### 2.7. Recycle Procedure of the Catalyst

After the reaction, the catalyst was separated from the reaction solution by centrifugation and then washed by CH_2_Cl_2_ (10 mL × 3), water (10 mL × 3), and ethanol (10 mL × 3) in sequence. After that, the catalyst was dried under a vacuum at 60 °C for 6 h. Then the recovered catalyst was used as the catalyst in subsequent catalytic reactions.

Considering the difficulty of recovering a small amount of catalyst, a simultaneous grouping experiment was performed. First, the feeding amount was increased by five times with 5.0 mg of catalyst added. After the first catalytic reaction, the catalyst was separated from the reaction solution by centrifugation, and then washed by CH_2_Cl_2_, water, and ethanol in turn. After that, the catalyst was dried under reduced pressure at 60 °C for 6 h. Then, from this, 1.0 mg catalyst was taken to catalyze the second round of reaction, and a simultaneous experiment continued using the remaining catalyst. After the second round of reaction and recovery, 1.0 mg catalyst was taken to catalyze the third round of reaction. Accordingly, experimental data were obtained for four consecutive rounds.

## 3. Results and Discussion

### 3.1. Synthesis and Characterization

**Scheme 1:**
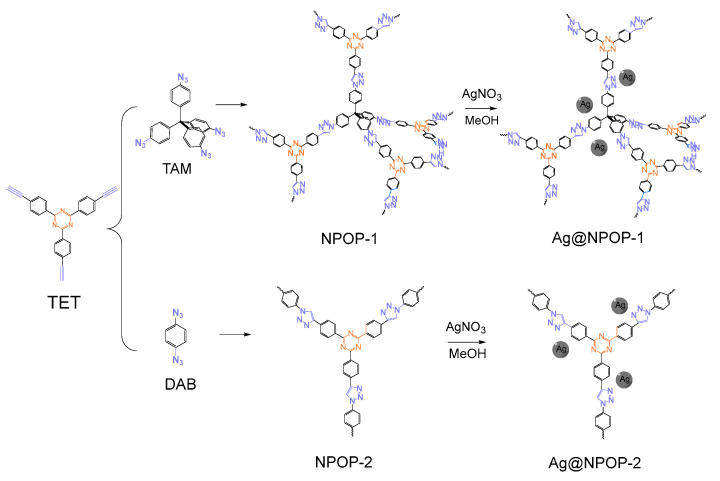
Illustrated synthetic procedures of NPOPs and Ag@NPOPs.

NPOP-1 and NPOP-2 were obtained by the reaction of 2,4,6-tris(4-ethynylphenyl)-1,3,5-triazine (TET) with tetrakis(4-azidophenyl)methane (TAM) and 1,4-diazidobenzene (DAB) via the CuAAC reaction (Scheme 1, Figures S1–S5). The 2,4,6-Tris(4-bromophenyl)-1,3,5-triazine was obtained under strong Brønsted acid conditions using 4-bromobenzonitrile [52]. The acetylene groups were introduced by a Pd-mediated Sonagashira coupling reaction to give the triazine compounds containing terminal acetylene groups [53,54]. Two compounds containing azide groups, TAM and DAB, were obtained by treatment of tetrakis(4-aminophenyl)methane or p-phenylenediamine with hydrazine hydrate under acetic acid conditions [55,56]. The CuAAC reaction was carried out in DMF at 100 °C for 24 h, using Cu(PPh_3_)_3_Br as the catalyst. Brownish-yellow powders NPOP-1 and NPOP-2 were obtained, which were insoluble in various organic solvents. Elemental analysis tests prove that the C, H, N contents were 66.72%, 4.56%, and 15.33% for NPOP-1 and 63.39%, 4.05%, and 22.31% for NPOP-2 (Table S1). Subsequently, Ag@NPOPs were obtained by wet impregnation and in situ reductions, where the NPOPs were first dispersed in AgNO_3_ aqueous solution, and afterward the adsorption and anchoring process of Ag^+^, methanol was used as the reducing agent to obtain Ag@NPOPs. The Ag content of Ag@NPOP-1 and Ag@NPOP-2 was 0.93 wt% and 1.29 wt%, respectively, determined by ICP.

**Figure 1:**
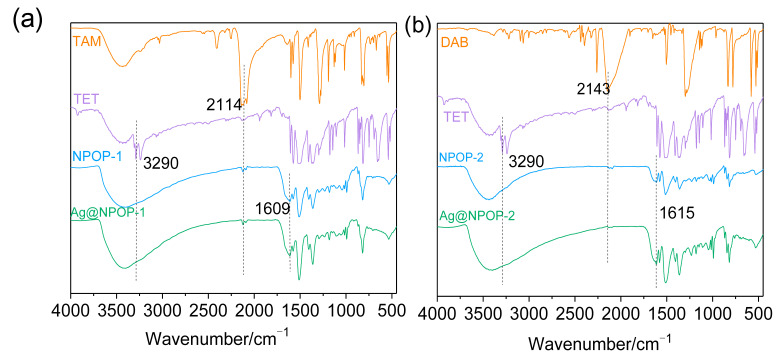
FT-IR spectra of: (**a**) TAM, TET, NPOP-1, and Ag@ NPOP-1, (**b**) DAB, TET, NPOP-2 and Ag@ NPOP-2.

The structures of NPOP-1 and NPOP-2 were identified by Fourier transform infrared (FT-IR) spectra (Figure 1). After the reaction, the characteristic peaks of azide groups at 2114 cm^−1^ and 2143 cm^−1^ for TAM and DAB attenuated significantly, and the terminal alkynyl-based characteristic absorption peak of TET at 3290 cm^−1^ disappeared [55]. In addition, the characteristic peak of a triazole ring was observed at 1609 cm^−1^ and 1615 cm^−1^ for NPOP-1 and NPOP-2, respectively, demonstrating the conducting of click reactions and the formation of a triazole ring [57]. Moreover, the characteristic absorption peaks of Ag@NPOP-1 and Ag@NPOP-2 have no obvious difference, which confirms that the chemical environment has no change after the embedding of Ag NPs. In particular, the FT-IR spectra are consistent with the original spectrum, and the characteristic peaks are retained after 24 h of immersion in the 6 mol L^−^^1^ HCl aqueous solution or 6 mol L^−^^1^ NaOH aqueous solution (Figure S6), proving the excellent chemical stability of NPOP-1 and NPOP-2.

**Figure 2:**
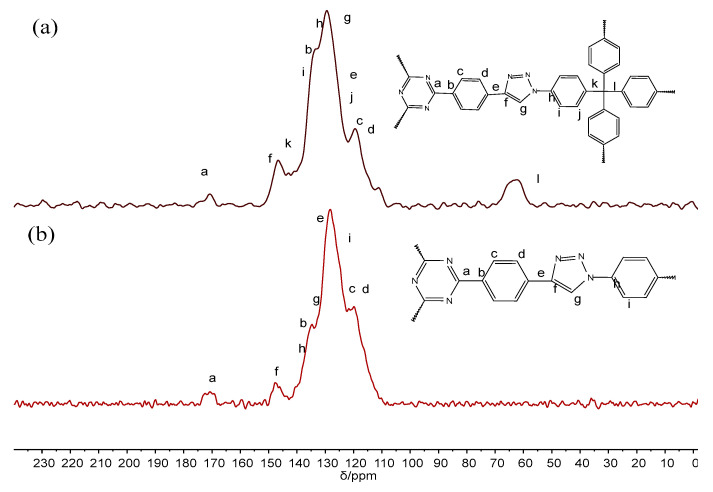
Solid-state ^13^C CP/MAS NMR spectra of: (**a**) NPOP-1, and (**b**) NPOP-2.

The chemical structure of the two NPOPs were further investigated using solid-state NMR solid-state ^13^C cross-polarized/magic-angle-spinning nuclear magnetic resonance (^13^C CP/MAS NMR) spectroscopy (Figure 2). The characteristic resonance peak at 170.7 ppm can be attributed to the sp^2^-hybridized carbon atom in the triazine ring [58]. The peaks at 147.6 ppm and 133.9 ppm originate from the two carbon atoms on the triazole ring, proving that the click reaction was conducted, which is further evidenced by the absence of the characteristic signal of alkyne carbon at 80 ppm. Additionally, the peak at 66.3 ppm in NPOP-1 corresponds to the alkane quaternary carbon in the precursor TAM, and the remaining peaks, in the range from 143 ppm to 110 ppm, are attributed to the phenyl carbon atoms [59].

**Figure 3:**
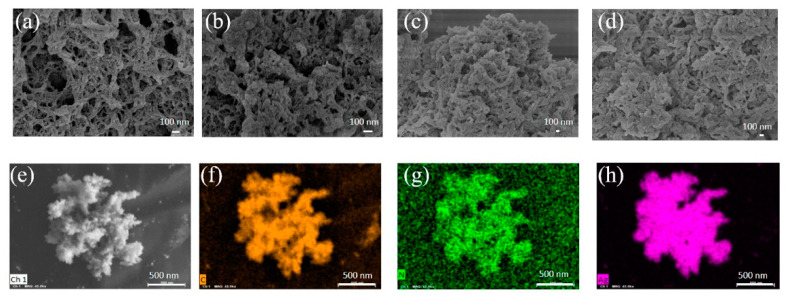
SEM images of: (**a**) NPOP-1, (**b**) Ag@NPOP-1, (**c**) NPOP-2, and (**d**) Ag@NPOP-2. The EDS elemental mappings of: (**e**) Ag@NPOP-1, (**f**) C, (**g**) N, and (**h**) Ag.

**Figure 4:**
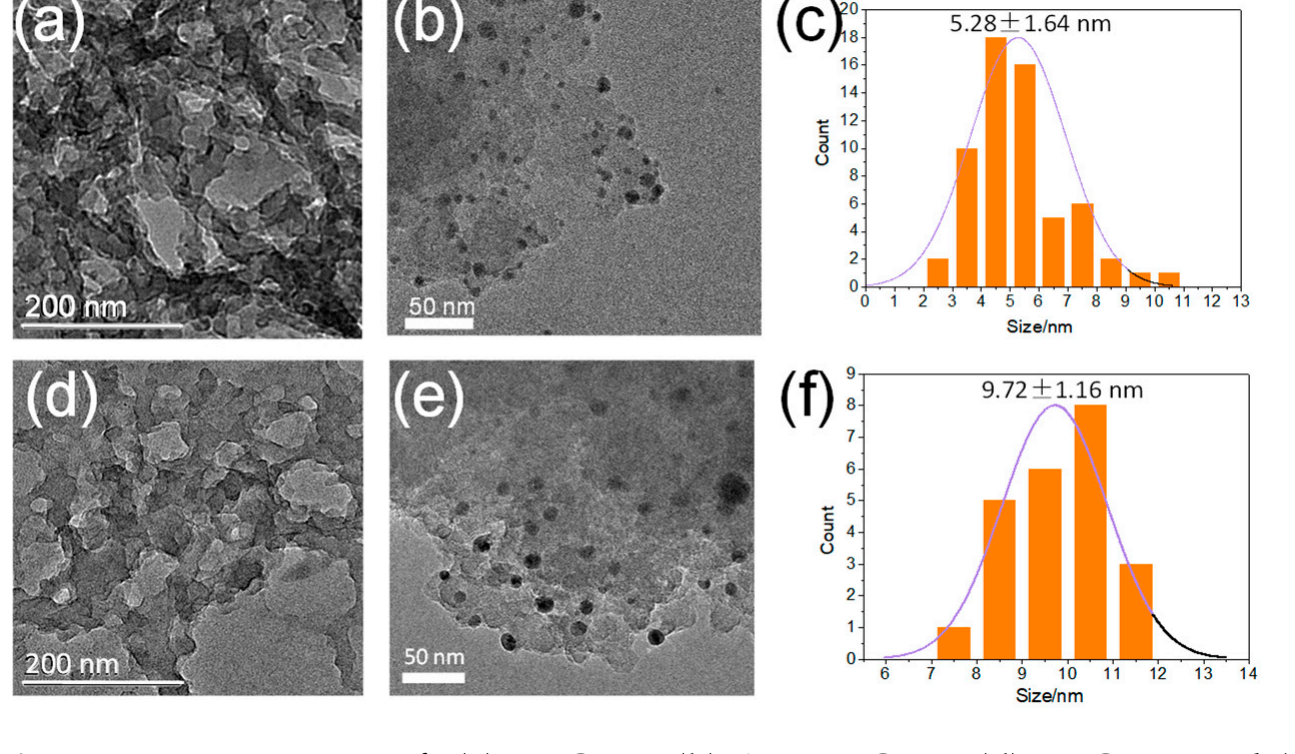
TEM images of: (**a**) NPOP-1, (**b**) Ag@NPOP-1, (**d**) NPOP-2, and (**e**) Ag@NPOP-2. Ag nanoparticle size distribution of: (**c**) Ag@NPOP-1, and (**f**) Ag@NPOP-2.

Scanner electron microscopy (SEM) and a transmission electron microscope (TEM) were employed to characterize the morphologies of NPOPs. The SEM images of NPOP-1 and NPOP-2 revealed randomly aggregated porous structures by tiny particles with disordered pores (Figure 3). NPOP-1 shows a fluffy and porous three-dimensional network structure, while NPOP-2 is made of tight randomly packed rod-shaped units. There is no significant change in the morphology of Ag@NPOPs, which proves that the anchoring of Ag nanoparticles does not alter the structure of NPOPs. Furthermore, EDS elemental distribution mappings of Ag@NPOP-1 manifest the uniform distribution of Ag, C, and N elements (Figure 3 and Figure S7). TEM images further present the porous structure of NPOPs; both NPOP-1 and NPOP-2 exhibit a highly cross-linked network-like structure (Figure 4). HR-TEM demonstrates that the Ag nanoparticles are uniformly distributed on the NPOPs’ substrates, the diameters of the Ag nanoparticles are 5.28 ± 1.64 nm and 9.72 ± 1.16 nm for Ag@NPOP-1 and Ag@NPOP-2, respectively. Apparently, Ag@NPOP-1 has much smaller Ag NPs.

Powder X-ray diffraction (PXRD) patterns show that NPOP-1 and NPOP-2 both exhibit broad diffraction peaks at around 20°, indicating the amorphous structure of NPOPs (Figure S8). In particular, the PXRD pattern of Ag@NPOP-1 shows no diffraction peaks of the Ag NPs, which may be attributed to the high dispersion and small particle size of the Ag NPs [60]. In comparison, the PXRD pattern of Ag@NPOP-2 shows a weak peak at 38°, corresponding to the Ag (111) crystal planes [61]. These results are consistent with the HR-TEM, indicating the smaller Ag NPs of Ag@NPOP-1. The thermogravimetric analysis displays that the weight loss below 100 °C is attributed to the volatilization of the remaining solvents. NPOP-1 has comparatively better thermal stability than NPOP-2 (Figure S9); NPOP-1 maintains thermal stability up to 230 °C, and NPOP-2 gradually decomposes above 100 °C.

**Figure 5:**
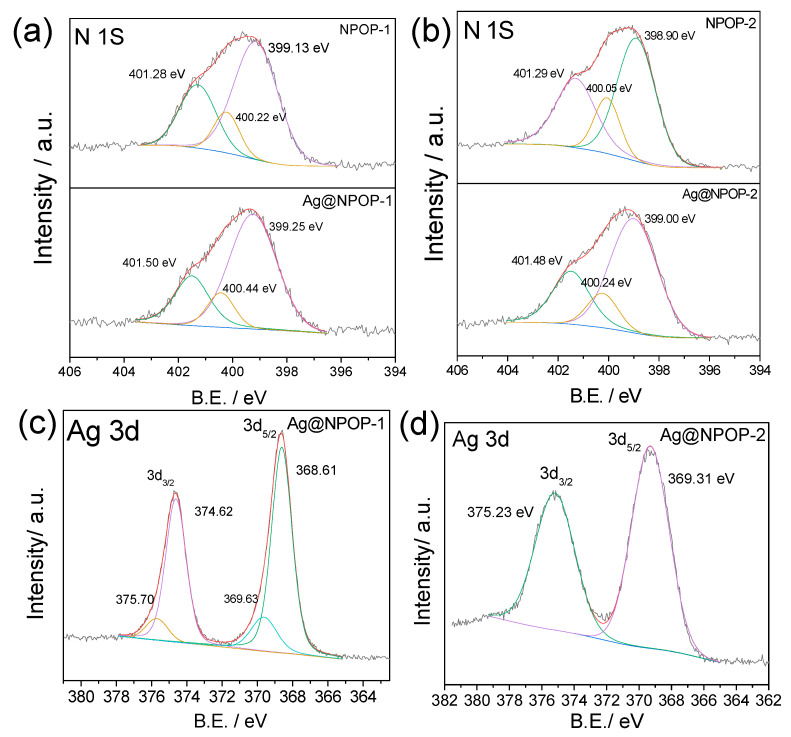
XPS and deconvoluted spectra of high-resolution N 1s for: (**a**) NPOP-1 and Ag@NPOP-1, and (**b**) NPOP-2 and Ag@NPOP-2. XPS and deconvoluted spectra of high-resolution Ag 3d for: (**c**) Ag@NPOP-1, and (**d**) Ag@NPOP-2.

The chemical states and interaction of Ag with N in the NPOPs were determined by XPS measurements (Figure 5 and Figure S10). The N 1s spectra of NPOPs can be deconvoluted into three peaks, appearing at: 401.28 eV, 400.22 eV, and 399.13 eV for NPOP-1, and 401.29 eV, 400.05 eV, and 398.90 eV for NPOP-2, which correspond to trizolic N=N, trizolicpyrrolic N, and triazine C=N, respectively [62]. After loading Ag nanoparticles, due to the coordination between N and Ag, the N 1s peaks have a positive shift. The trizolic N=N and trizolicpyrrolic N in Ag@NPOP-1 have a 0.22 eV shift toward the higher BE filed in comparison with a 0.12 eV shift of triazine C=N. Similarly, Ag@NPOP-2, trizolic N=N, and trizolicpyrrolic N have a 0.19 eV shift toward the higher BE filed in comparison with a 0.10 eV shift of triazine C=N. This indicates that stronger coordination interactions exist between Ag and the triazole ring. The XPS spectra of the Ag 3d region of Ag@NPOPs further revealed the presence of Ag species. The double binding energy signals, at 374.62 eV and 368.61 eV for Ag@NPOP-1, and at 375.23 eV and 369.31 eV for Ag@NPOP-2, are attributed to the 3d_3/2_ and 3d_5/2_ binding energies of the Ag(0) peaks, respectively [63].In addition, the signals existing at 375.70 eV and 369.63 eV for Ag@NPOP-1 are 3d_3/2_ and 3d_5/2_ of Ag(δ+) species, which may be due to partial oxidation of the material during preparation and testing.

**Figure 6:**
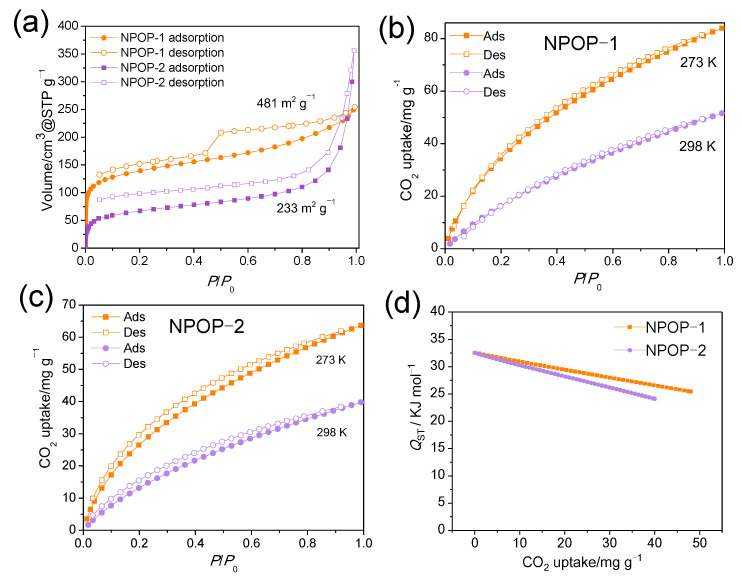
(**a**) N_2_ adsorption–desorption isotherms of NPOP-1 and NPOP-2 at 77 K. CO_2_ adsorption–desorption isotherms, at 273 K and 298 K, of: (**b**) NPOP-1, (**c**) NPOP-2, and (d) isosteric heats of CO_2_ adsorption of NPOP-1 and NPOP-2 calculated with the virial method.

The porosity of the NPOP-1 and NPOP-2 were investigated by N_2_ isothermal adsorption-desorption measurements at 77 K. As shown in Figure 6, NPOP-1 displays a representative type IV adsorption isotherm, and NPOP-2 exhibits a typical type I isotherm. The N_2_ adsorption-desorption isotherms of both the NPOPs manifest a rapid growth of N_2_ uptake in a low relative pressure range *P*/*P*_o_ < 0.01, demonstrating the existence of micropores. The presence of mesopores is evidenced by the hysteresis loop that accompanies the desorption curve. Moreover, the sharp increase of N_2_ uptake after *P*/*P*_o_ > 0.9 in the adsorption isotherm of NPOP-2 indicates the presence of macropores. The Brunauer-Emmett-Teller (BET) specific surface areas of NPOP-1 and NPOP-2 are calculated to be 481 and 233 m^2^ g^−1^, respectively, as shown in Table S2. The total pore volumes of NPOP-1 and NPOP-2 measured at *P*/*P*_o_ = 0.99 are 0.39 and 0.55 cm^3^ g^−1^, respectively. The micropore volumes are 0.23 cm^3^ g^−1^ for NPOP-1 and 0.11 cm^3^ g^−1^ for NPOP-2, accounting for 59.0% and 20.0% of the total pore volumes, respectively. The higher specific surface area of NPOP-1 probably results from the rigid steric structure of the precursor TAM, which qualifies the permanent pore structure and high porosity seen in NPOP-1. Nevertheless, the higher total pore volume of NPOP-2 may be attributed to its partial macropores. Furthermore, the BJH and Horvath-Kawazoe models are used to evaluate the pore size distribution of NPOPs. The BJH model indicates that no macropores exist in NPOP-1, while a wide distribution range of macropores and mesopores from 20–80 nm are shown in NPOP-2 (Figure S11). The Horvath-Kawazoe model is used to explore the micropore distribution of NPOPs. The results show that the micropore distribution plot of NPOP-1 shows a predominant peak at 0.82 nm, and the micropores of NPOP-2 are mainly concentrated in the range of 1.19–3 nm. It is worth mentioning that the specific surface areas of the NPOPs are lower than the related materials, which may be attributed to the high catalytic activity of Cu(PPh_3_)_3_Br [64,65,66]. This leads to the rapid generation of a low-polymerized framework with few overlapping parts, and the remaining precursors connect randomly resulting in the formation of an unregulated framework structure.

Considering the high porosity and abundant N content of NPOPs, we further investigated their CO_2_ adsorption performance. As shown in Figure 6, at 273 K and 0.1 MPa, the CO_2_ adsorption capacity is 84.0 mg g^−1^ for NPOP-1 and 63.7 mg g^−1^ for NPOP-2. At 298 K and 0.1 MPa the CO_2_ adsorption capacities of NPOP-1 and NPOP-2 are 51.6 and 39.8 mg g^−1^, respectively. The higher CO_2_ uptake value of NPOP-1 may be attributed to its higher specific surface area and abundant microporous content. Both the NPOPs exhibit competitive CO_2_ adsorption values compared to related triazine-/triazole-based porous organic polymers reported in the literature (Table S3) [65,66,67,68,69,70,71,72,73,74,75,76,77,78,79,80,81,82,83]. To further understand the interaction between CO_2_ and NPOPs, the CO_2_ isosteric adsorption heats (*Q*_st_) are calculated based on the Clausius-Clapeyron equation using the CO_2_ adsorption isotherms at 273 K and 298 K (Figure 6, Figures S12 and S13). The *Q*_st_ values of NPOPs both decrease with the increase in CO_2_ adsorption amounts. At zero coverage, the *Q*_st_ values of NPOP-1 and NPOP-2 are calculated to be 32.56 and 32.51 kJ mol^−1^, respectively. The *Q*_st_ values are higher than other porous adsorbents and triazine-/triazole-based porous organic polymers (Table S3) [84,85]. Higher *Q*_st_ values suggest stronger interactions between NPOPs and CO_2_; moreover, the NPOP-1 shows a larger *Q*_st_ than NPOP-2 at the same absorption value, implying a stronger affinity between CO_2_ and NPOP-1. The high CO_2_ uptake may originate from the large specific surface area and abundance of the electron-rich N element of NPOPs [86].

### 3.2. Catalytic Activity of Ag@NPOPs towards CO_2_ Conversion

#### 3.2.1. Carboxylative Cyclization of Propargylic Amines with CO_2_

The conversion of CO_2_ into high-value chemicals under mild conditions (atmospheric pressure) is a promising approach to reducing the greenhouse effect and saving valuable fossil energy. Among these promising reaction pathways, the carboxylative cyclization reaction of CO_2_ with propargylamine to 2-oxazolidinone is representative; moreover, oxazolidinones have been widely used as chemical intermediates and pharmaceuticals [87,88]. It is of vital importance to explore the mild reaction conditions and the higher reaction efficiency. Considering that the NPOPs have a high adsorption capacity for CO_2_, Ag@NPOPs were used as the catalysts in the carboxylative cyclization reaction of CO_2_ with propargylamine. The *N*-benzylprop-2-yn-1-amine [89] was used as a model substrate to study optimal reaction conditions (Figures S14 and S15), and the results of various controlled experiments are shown in Table 1. When CH_3_CN was used as a solvent and DBU as a base, the yield of 2-oxazolidinone was 97.0% for Ag@NPOP-1 with a TOF value of 1125.1 h^−1^, and 93.0% for Ag@NPOP-2 with a TOF value of 777.7 h^−1^ (Table 1, Entry 2, 3). The Ag@NPOP-1 shows a higher catalytic efficiency because of its larger specific surface area, higher CO_2_ adsorption capability, and smaller Ag NPs size. No product was detected when NPOP-1 or NPOP-2 were used as catalysts, which proves the vital role of Ag NPs in the catalysis reaction (Table 1, Entry 4, 5). Similarly, no product was detected in the absence of DBU, proving the essential role of DBU as a base. Additionally, the catalytic efficiency in different solvents was investigated, and the product yields in DMSO, DMF, and EtOH were 58%, 42%, and 15%, respectively (Table 1, Entry 7–9). Therefore, CH_3_CN is the optimal solvent. In addition to DBU, other different common bases were also adopted to explore the catalytic effect, when we replaced DBU with Cs_2_CO_3_ and K_2_CO_3_, the 2-oxazolidinone yields were only 4.0% and 2.0%, respectively, in CH_3_CN. When NaOH was used as a base, no product was detected. The influence of temperature in the reaction was also investigated. At lower temperatures, the product yields were significantly reduced as a result of inadequate reactivity. When the reaction temperatures were 40 °C and 30 °C, the product yields of 2-oxazolidinone were 86.0% and 40.0%, respectively.

#### 3.2.2. Carboxylation of Phenylacetylene with CO_2_

The carboxylation of terminal alkynes to phenylpropiolic acid is another feasible way to realize the high-value transformation of CO_2_. Furthermore, it accords with the atomic economy concept and is more environmentally friendly compared to traditional methods. Propiolic acid is a valuable intermediate utilized in pharmaceuticals and fine chemicals [90,91]. We chose 1-ethynylbenzene as a model substrate (Figure S16), and the results of various controlled experiment are shown in Table 2. When Ag@NPOP-1 was used as the catalyst and the reaction time was 12 h at 60 °C with 3 eq. Cs_2_CO_3_, the yield of 3-phenylproparylic acid reached 94.0%, slightly higher than that of Ag@NPOP-2 (92.1%) (Table 2, Entry 1, 2). Surprisingly, the reaction also proceeded with metal-free NPOP-1 and NPOP-2, but the product yield was only 55.4% and 51.2%, respectively (Table 2, Entry 3, 4), which suggests that the nitrogen atoms in the triazole and triazine rings act as active sites to promote CO_2_ conversion. By increasing the amount of catalyst from 2.0 mg to 5.0 mg, the yield of 3-phenylpropargylic acid was 94.2%, proving that a larger amount of catalyst could not significantly facilitate the catalytic efficiency. Subsequently, several solvents were screened: DMF gave a medium yield of 73.7%, and the product yields in MeCN and EtOH were much lower at 11.9% and 3.4%, respectively (Table 2, Entry 6–8). Accordingly, DMSO is the best solvent for this reaction because Cs_2_CO_3_ has a higher solubility in DMSO and it also works as a good solvent for CO_2_ due to its higher polarity. In addition to Cs_2_CO_3_, other bases were tested. When K_2_CO_3_, DBU, or NaOH were used as a base, the yield of 3-phenylproparylic acid was 11.5%, 51.0%, and 4.3%, respectively (Table 2, Entry 9–11), suggesting that Cs_2_CO_3_ is the preferable base. Apart from that, the influence of different base dosages in the reaction was explored. When Cs_2_CO_3_ was 2 eq. and 1 eq., the corresponding product yield was 60.8% and 24.5%, respectively. The reduction in the amount of base was followed by a reduction in the product yield. It shows the base’s essential role in the conversion of CO_2_, as it helps the deprotonation process of phenylacetylene to facilitate the formation of reaction intermediates. Additionally, the impact of temperature on the reaction was also investigated, and reducing temperature led to a notably decreased yield, when the reaction was conducted at 50 °C and 40 °C, the product yield was 65.2% and 18.0%, respectively.

### 3.3. Catalyst Stability

**Figure 7:**
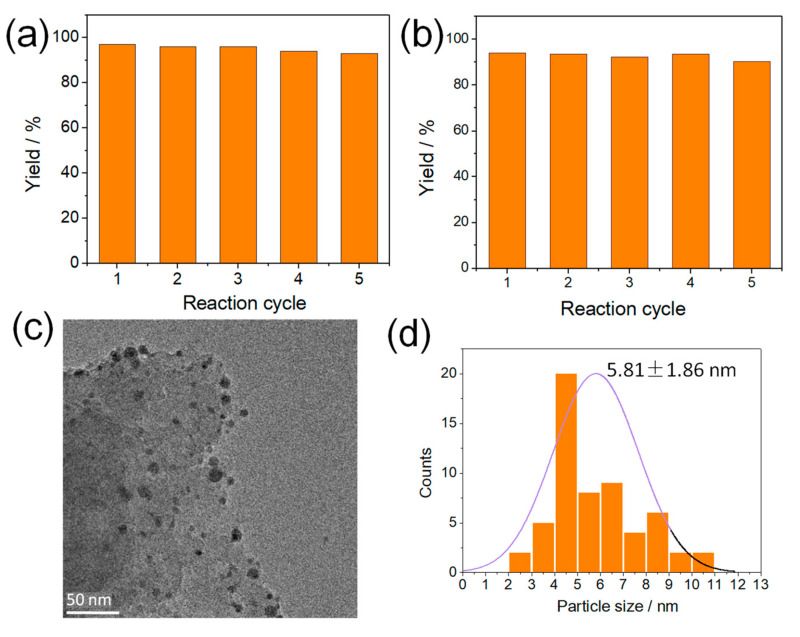
Recycling performance of Ag@NPOP-1 displaying: (**a**) carboxylative cyclization of propargylic amines, (**b**) carboxylation of phenylacetylene with CO_2_, (**c**) HR-TEM image, and (**d**) Ag particle size distribution of Ag@NPOP-1 after reuse for five cycles.

Recycling tests were conducted to confirm the reusability and endurance of Ag@NPOP-1 toward the carboxylative cyclization of propargylic amines and the carboxylation of phenylacetylene with CO_2_ (Figure 7a,b). After being used for five times, the yield of 2-oxazolidinone and phenylpropionic acid were 93.0% and 90.4%, respectively, with no distinct loss of catalytic efficiency. TEM of reused Ag@NPOP-1 (Figure 7c) shows that the Ag NPs remain uniformly dispersed on the NPOP substrate with slight agglomeration, with an average particle diameter of 5.7 nm. The FT-IR and PXRD (Figures S17 and S18) of Ag@NPOP-1 show that there is no significant change after it is used five times, proving the excellent reusability of Ag@NPOP-1.

### 3.4. Catalytic Mechanism

The catalytic efficiency of Ag@NPOPs is compared with the reported data. As shown in Tables S4 and S5, Ag@NPOPs exhibit highly competitive catalytic activity for both the CO_2_ conversion reactions and the TOF values of Ag@NPOPs are much higher than those of most reported catalytic systems [16,17,63,92,93,94,95,96,97,98,99,100,101,102,103,104,105,106,107,108,109,110]. The excellent catalytic activity is attributed to the following three aspects: (1) Ag@NPOP-1 contains two kinds of nitrogen heterocycles, triazine and triazole rings, which can absorb and enrich CO_2_ and can anchor Ag NPs as well; (2) The abundant micropores and high specific surface area of NPOP-1 endow it an outstanding affinity and adsorption capacity for CO_2_; (3) The high dispersion and small particle size of Ag NPs in Ag@NPOP-1 further enhance its catalytic activity.

**Scheme 2:**
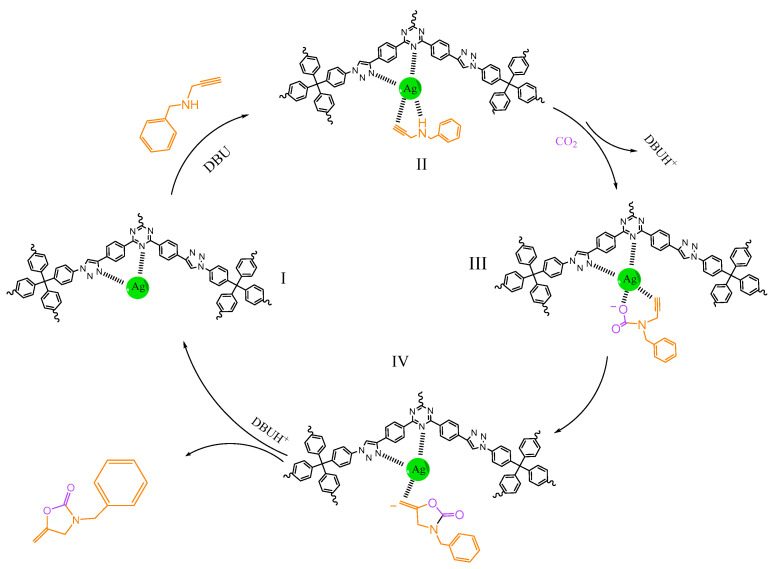
Proposed catalytic mechanism of carboxylative cyclization of propargylic amines with CO_2_ catalyzed by Ag@NPOP-1.

Based on the above observations and previous reports [100,111], a possible catalytic mechanism of carboxylative cyclization of propargylic amines with CO_2_ is proposed (Scheme 2). When propargylamine enters the pores of NPOP-1, the amino and carbon-carbon triple bonds in propargylamine interact with Ag NPs, further leading to the activation of hydrogen protons on the amino group, and, with the assistance of DBU, the CO_2_ molecule adsorbed by NPOP-1 attacks the amino group to produce a carbamate intermediate (III). Subsequently, the negatively charged oxygen attacks the carbon-carbon triple bond, resulting in an intramolecular cyclization of propargylamine with the formation of a negatively charged carbon-carbon double bond (IV). After the proton from DBUH^+^ is seized, the final 2-oxazolidinone product is generated.

**Scheme 3:**
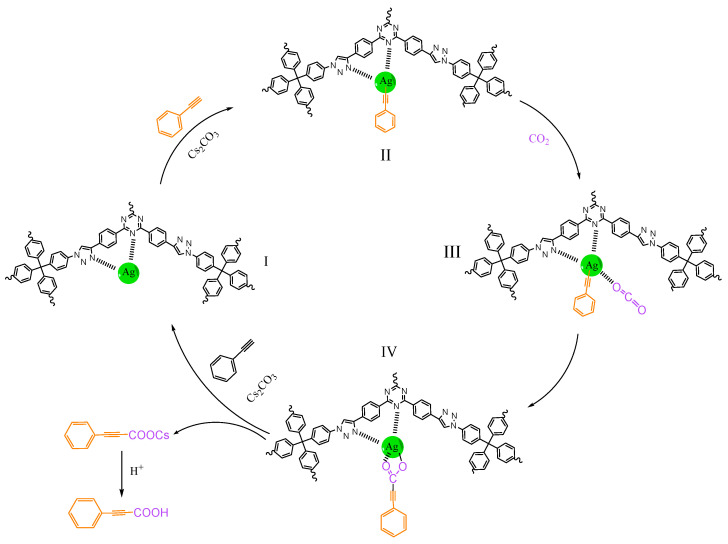
Proposed catalytic mechanism of carboxylation of terminal alkynes with CO_2_ catalyzed by Ag@NPOP-1.

Apart from that, the mechanism of the carboxylation of terminal alkynes is postulated (Scheme 3) [112,113]. First, the terminal alkynes enter the pores of NPOP-1 and are deprotonated by the immobilized Ag NPs with the assistance of Cs_2_CO_3_, forming the Ph-C≡C-Ag@NPOP-1 intermediate (II). Subsequently, the CO_2_ molecule enriched by adsorption of abundant nitrogen atoms in the triazine and triazole rings attacks the adjacent nucleophilic active alkyne carbon and further inserts into the C-Ag bond to form a cesium carboxylate species (IV). In the presence of Cs_2_CO_3_ and nearby terminal alkynes, the cesium carboxylate detaches from the Ag NPs and enters the solvent, where Ag@NPOP-1 is regenerated and adsorbs new terminal alkynes for the next round of catalysis. Eventually, the carboxylate is acidified by hydrochloric acid to obtain propargylic acid.

## 4. Conclusions

In summary, we have synthesized organic porous polymers NPOP-1 and NPOP-2 functionalized with triazine and triazole nitrogen heterocycles by the click reaction. The NPOPs are porous with specific surface areas of 481 m^2^ g^−1^ for NPOP-1 and 223 m^2^ g^−1^ for NPOP-2. The abundant nitrogen content confers high CO_2_ affinity and adsorption capacity on the NPOPs with the CO_2_ adsorption capacity of NPOP-1 and NPOP-2 of 84.0 and 63.7 mg g^−1^, respectively, at 273 K and 1 atm. The Ag@NPOPs are obtained by simple wet impregnation and in situ reductions with highly dispersed small-size Ag nanoparticles. The Ag@NPOPs show excellent catalytic activity in the catalysis of the high-value conversion of CO_2_ with propargylamine and terminal alkynes, with the highest TOF values reaching 1125.1 h^−1^ and 90.9 h^−1^, respectively. The Ag@NPOP-1 shows higher catalytic efficiency because of its larger specific surface area, higher CO_2_ adsorption capability, and smaller Ag NPs size. The Ag@NPOPs show excellent catalytic stability and durability with no significant decrease in catalytic activity after five consecutive cycles. More than demonstrating a dual-functional CO_2_ conversion catalyst, this work provides some new inspirations for the design and construction of novel multifunctional catalysts.

## Figures and Tables

**Table 1:** Catalysis of NPOPs and Ag@NPOPs for the carboxylative cyclization of propargylic amines with CO_2_ ^a^.

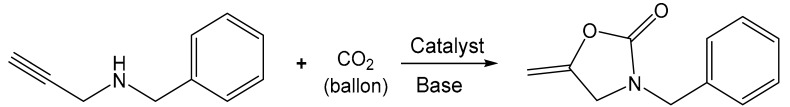							
Entry	Catalyst	Solvent	*T*/°C	Base	Time/h	Yield/%	TOF/h^−1^
1	\	CH_3_CN	50	DBU	2	N.A.	
2	Ag@NPOP-1	CH_3_CN	50	DBU	2	97.0	1125.1
3	Ag@NPOP-2	CH_3_CN	50	DBU	2	93.0	777.6
4	NPOP-1	CH_3_CN	50	DBU	2	N.A.	
5	NPOP-2	CH_3_CN	50	DBU	2	N.A.	
6	Ag@NPOP-1	CH_3_CN	50	\	2	N.A.	
7	Ag@NPOP-1	DMSO	50	DBU	2	58	672.7
8	Ag@NPOP-1	DMF	50	DBU	2	42	487.2
9	Ag@NPOP-1	EtOH	50	DBU	2	15	174.0
10	Ag@NPOP-1	CH_3_CN	50	Cs_2_CO_3_	2	4.0	46.4
11	Ag@NPOP-1	CH_3_CN	50	K_2_CO_3_	2	2.0	23.2
12	Ag@NPOP-1	CH_3_CN	50	NaOH	2	N.A.	
13	Ag@NPOP-1	CH_3_CN	40	DBU	2	86.0	997.5
14	Ag@NPOP-1	CH_3_CN	30	DBU	2	40.0	464.0

^a^ Reaction conditions: Propargylic amine (0.2 mmol), Ag@NPOP-1 or Ag@NPOP-2 (1.0 mg), base (0.1 mmol), CO_2_ (balloon), solvent (1.0 mL). The reaction mixture was stirred at 50 °C for 2 h. Yield was calculated by ^1^H NMR with 1,4-dinitrobenzene as internal standard.

**Table 2:** Catalysis of NPOPs and Ag@NPOPs for the carboxylation of phenylacetylene with CO_2_ ^a^.

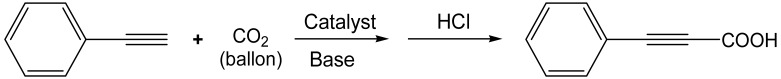							
Entry	Catalyst	Solvent	*T*/°C	Base (Amount/mmol)	Time /h	Yield/%	TOF /h^−1^
1	Ag@NPOP-2	DMSO	60	Cs_2_CO_3_(0.6)	12	92.1	64.2
2	Ag@NPOP-1	DMSO	60	Cs_2_CO_3_(0.6)	12	94.0	90.9
3	NPOP-2	DMSO	60	Cs_2_CO_3_(0.6)	12	51.2	
4	NPOP-1	DMSO	60	Cs_2_CO_3_(0.6)	12	55.4	
5	Ag@NPOP-1 ^b^	DMSO	60	Cs_2_CO_3_(0.6)	12	94.2	36.4
6	Ag@NPOP-1	DMF	60	Cs_2_CO_3_(0.6)	12	73.7	71.2
7	Ag@NPOP-1	ACN	60	Cs_2_CO_3_(0.6)	12	11.9	11.5
8	Ag@NPOP-1	EtOH	60	Cs_2_CO_3_(0.6)	12	3.4	3.3
9	Ag@NPOP-1	DMSO	60	K_2_CO_3_(0.6)	12	11.5	11.1
10	Ag@NPOP-1	DMSO	60	DBU(0.6)	12	51.0	49.3
11	Ag@NPOP-1	DMSO	60	NaOH (0.6)	12	4.3	4.2
12	Ag@NPOP-1	DMSO	60	Cs_2_CO_3_(0.4)	12	60.8	58.8
13	Ag@NPOP-1	DMSO	60	Cs_2_CO_3_(0.2)	12	24.5	23.7
14	Ag@NPOP-1	DMSO	50	Cs_2_CO_3_(0.6)	12	65.2	63.0
15	Ag@NPOP-1	DMSO	40	Cs_2_CO_3_(0.6)	12	18.0	17.4

Reaction conditions: (^a^) Phenylacetylene (0.2 mmol), catalyst (2.0 mg), CO_2_ (balloon), solvent (1.0 mL). The reaction mixture was stirred at 60 °C for 12 h. Yield was calculated by ^1^H NMR. (^b^): catalyst, 5.0 mg.

## Data Availability

Not applicable.

## Supplementary Materials

The following supporting information can be downloaded at: https://www.mdpi.com/article/10.3390/nano12183088/s1, Additional experimental details, Figures S1–S18: (NMR, IR, EDS, XPS spectra, PXRD, TG, pore size, adsorption data, and Tables S1–S5: (elemental analysis, porosity, CO_2_ absorption, and catalysis data)).
