# Supplementary material for: Nitrogen-Rich Porous Organic Polymers with Supported Ag Nanoparticles for Efficient CO2 Conversion
Source: Nanomaterials (Basel). 2022 Sep 6;12(18):3088. doi: 10.3390/nano12183088 (PMC9501012; doi:10.3390/nano12183088)
Supplement: Supplementary file 1 [file nanomaterials-12-03088-s001.zip › nanomaterials-1879389-supplementary.pdf]

nanomaterials

MDPI

Supporting Information

Nitrogen-Rich Porous Organic Polymers with Supported Ag Nanoparticles for Efficient CO_2 Conversion

Jinyi Wu [1], Shasha Ma [1], Jiawei Cui [1], Zujin Yang [2] and Jianyong Zhang [1,*]

1 MOE Laboratory of Polymeric Composite and Functional Materials, School of Materials Science and Engineering, Sun Yat-sen University, Guangzhou 510275, China
2 School of Chemical Engineering and Technology, Sun Yat-sen University, Guangzhou 510275, China
* Correspondence: zhjyong@mail.sysu.edu.cn

Materials and Characterization

All reagents and solvents were purchased from commercial suppliers and used as received without further purification unless otherwise stated. ^{1}H NMR spectra were recorded on a Bruker Avance III/400 (400 MHz) spectrometer, and the spectral data were processed with Mestrenova software. FTIR spectra were recorded on a LIFM Frontier spectrometer in KBr pellets in the range 4000-400 cm^{-1}. Scanning electron micrographs were recorded on a JSM6330F emission environmental scanning electron microscope. Samples were prepared by dispersing in EtOH upon sonication and placing on top of aluminum foil. TEM were conducted on a FEI Tecnai G2 Spirit 300 kV transmission electron microscope. Samples were prepared by dispersing in EtOH upon sonication and placing on top of the carbon grid. N_2 adsorption measurements were performed using a Quantachrome Autosorb-iQ2 analyzer. Prior to analysis, the sample was degassed at 80 °C for 24 h to remove solvated molecules. XRD measurement was performed on D-MAX 2200 VPC instrument. Thermogravimetric analyses were carried out in a nitrogen stream using Netzsch STA 449F3-jupiter thermogravimetric-differential scanning calorimetry (TG-DSC) analyzer with a heating rate of 10 °C min^{-1}. X-ray photoelectron spectroscopy (XPS) was tested on Nexsa Thermo ESCALAB 250Xi instrument with an Al Kα X-ray source (Thermo Fisher). Metal analyses were performed by using a Thermo Scientifi iCAP RQ ICP-MS instrument or HITACHI Z-2000 atomic absorption spectrophotometer. Before analysis, the material was digested by aqua regia at 80 °C for 12 h.

Synthesis of 1,4-Diazidobenzene

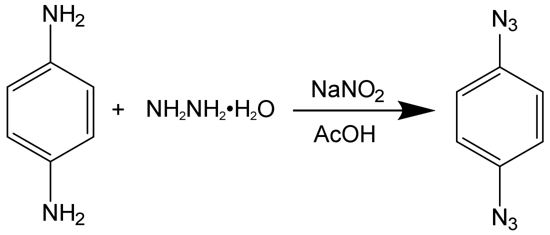

Synthesized according to the previously reported procedure [1]. Under nitrogen atmosphere, *p*-phenylenediamine (1.297 g, 12.0 mmol) and $NaNO_2$ (1.656 g, 24.0 mmol) was mixed in CH_2Cl_2 (60 mL). After that, AcOH (5.4 mL, 96.0 mmol) and hydrazine hydrate (2.92 mL, 60.0 mmol) was added. The brown solution mixture was stirred at RT for 1 h. After the reaction was finished, distilled water (40 mL) was added to the solution and the reaction mixture was extracted for three times. The organic solvent was removed under reduced pressure. The crude product was subjected to silica gel chromatography with petroleum ether as eluent (0.293 g, 19% yield). ^{1}H NMR (400 MHz, $CDCl_3$): δ 7.04 (s, 4H).

Synthesis of Tetrakis(4-azidophenyl)methane [2]

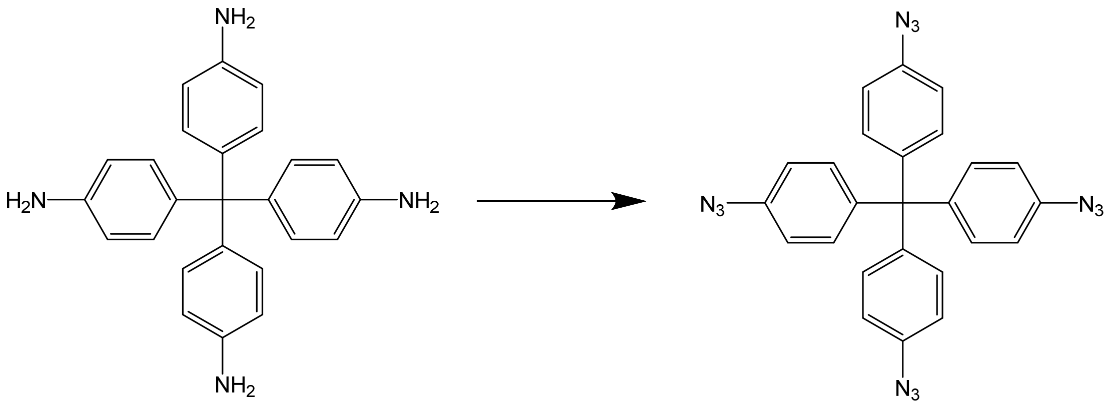

Under nitrogen atmosphere, tetrakis(4-aminophenyl)methane (0.6 g, 1.6 mmol) and $NaNO_2$ (0.44 g, 24.0 mmol) was mixed in CH_2Cl_2 (60 mL). After that AcOH (1.5 mL, 25.6 mmol) and hydrazine hydrate (0.8 mL, 16.0 mmol) was added. The brown solution mixture was stirred at RT for 1 h. After the reaction was finished, distilled water (40 mL) was added to the solution and the reaction mixture was extracted for three times. The organic solvent was removed under reduced pressure. The crude product was subjected to silica gel chromatography with petroleum ether as eluent (0.27 g, 27.6 % yield). ^{1}H NMR (400 MHz, $CDCl_3$): δ 7.15 (d, J = 8.7 Hz, 8H), 6.96 (d, J = 8.7 Hz, 8H).

4. Synthesis of 2,4,6-tris(4-bromophenyl)-1,3,5-triazine

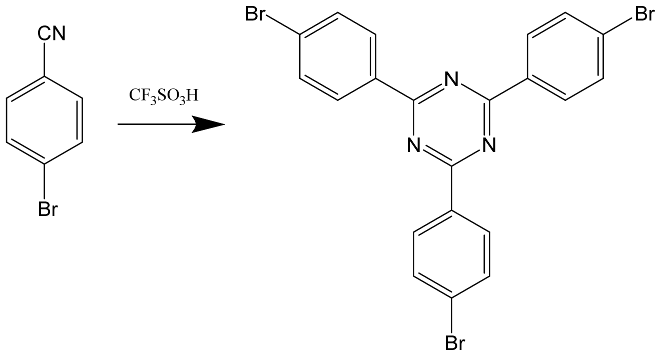

Synthesized according to the previously reported procedure [3]. 4-Bromobenzonitrile (3.0 g, 16.5 mmol) was slowly added to trifluormethane sulfonic acid (2.7 mL, 30 mmol). The mixture was stirred at 25 °C for 24 h and afterwards 60 mL deionised water was added. Then the solid was filtered, and washed with deionized water (40 mL × 3) and acetone (10 mL × 2). The solid was dried under vacuum at 60 °C to give 2,4,6-tris(4-bromophenyl)-1,3,5-triazine as a white solid (2.95 g, 98%). ^{1}H NMR (400 MHz, $CDCl_3$): δ 8.63 (d, J = 8.6 Hz, 6H), 7.74 (d, J = 8.6 Hz, 6H).

Synthesis of 2,4,6-tris(4-((trimethylsilyl)ethynyl)-phenyl)-1,3,5-triazine

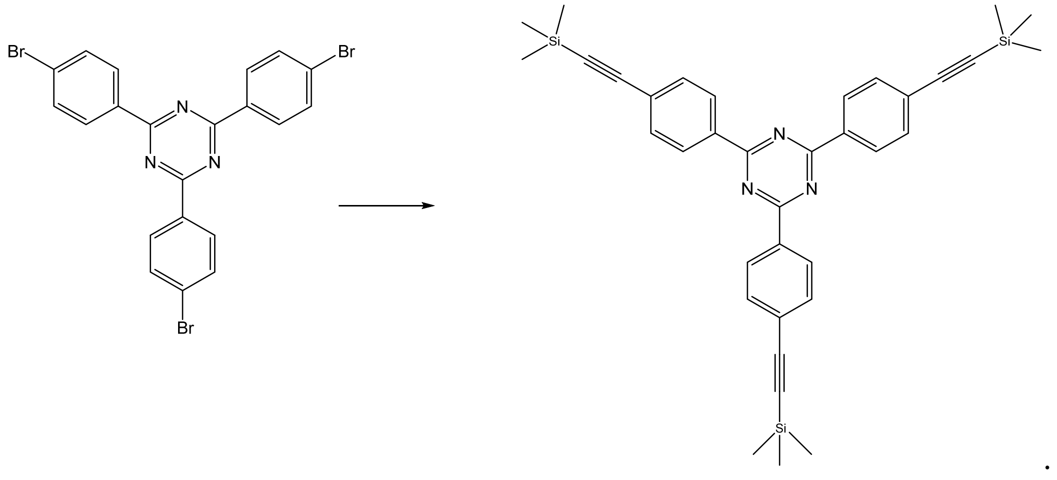

Synthesized according to the previously reported procedure [4]. Under N_2 atmosphere, 2,4,6-tris(4-bromophenyl)-1,3,5-triazine (2.8 g, 5.12 mmol), $PdCl_2(PPh_3)_2$ (0.230 g, 0.26 mmol), CuI (46.0 mg, 0.26 mmol), and PPh_3 (130.0 mg, 0.49 mmol) were mixed in anhydrous iPr_2NH (80 mL). Trimethylsilylacetylene (7.1 mL, 51.2 mmol) was added via a syringe. The reaction mixture was brought to reflux (90 °C) for 24 h and then cooled down to room temperature. The solvent was removed under reduced pressure, and $CHCl_2$ was added to dissolve the residue. The mixture was filtered through a pad of celite, the filtrate was washed with dilute Na_2EDTA solution and then dried over anhydrous Na_2SO_4. The solution was concentrated, and ethanol was added to obtain a white solid product (2.02 g, 71.3% yield). ^{1}H NMR (400 MHz, $CDCl_3$): δ 8.71 (d, *J* = 8.4 Hz, 6H), 7.67 (d, *J* = 8.4 Hz, 6H), 0.32 (s, 27H).

Synthesis of 2,4,6-tris(4-ethynylphenyl)-1,3,5-triazine

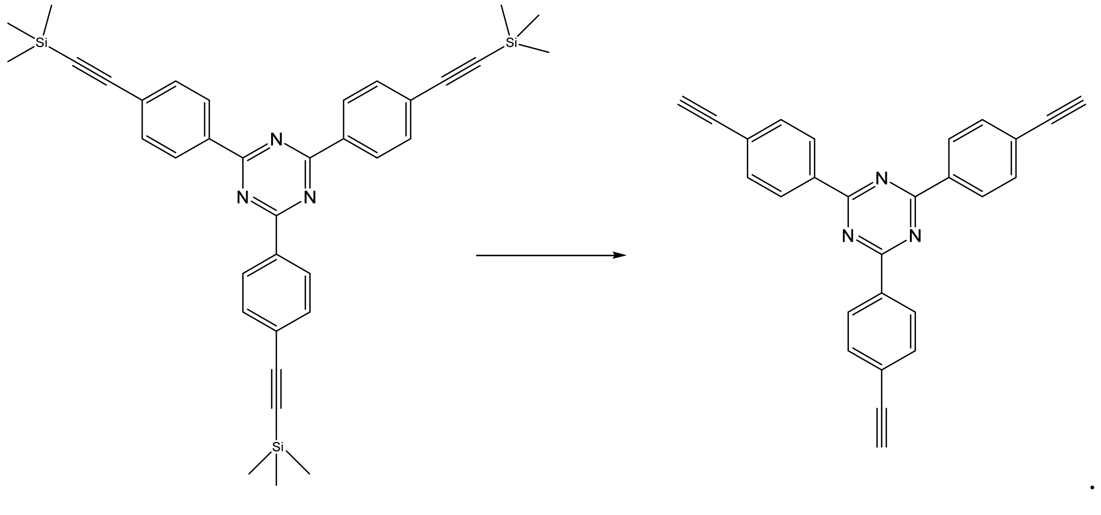

Synthesized according to the previously reported procedure [5]. A solution of NaOH (1.337 g, 33.4 mmol) in CH_3OH (15 mL) was added to a solution of 2,4,6-tris(4-((trimethylsilyl)ethynyl)-phenyl)-1,3,5-triazine (2.0 g, 3.3 mmol) in CH_2Cl_2 (60 mL), and the resulting mixture was stirred for 12 h at RT. The reaction mixture was washed with water, the aqueous phase was extracted with CH_2Cl_2. The combined organic phase was washed with brine, and then dried over anhydrous Na_2SO_4. The solvent was removed under reduced pressure, and the raw product was subjected to silica gel chromatography with petroleum ether/ethyl acetate (*v*:*v* 5:1) as eluent. The product was obtained as yellow solid (0.85 g, 67.3% yield). ^{1}H NMR (400 MHz, $CDCl_3$): δ 8.74 (d, *J* = 8.2 Hz, 6H), 7.71 (d, *J* = 8.2 Hz, 6H), 3.31 (s, 3H).

Synthesis of *N*-propargylbenzylamine

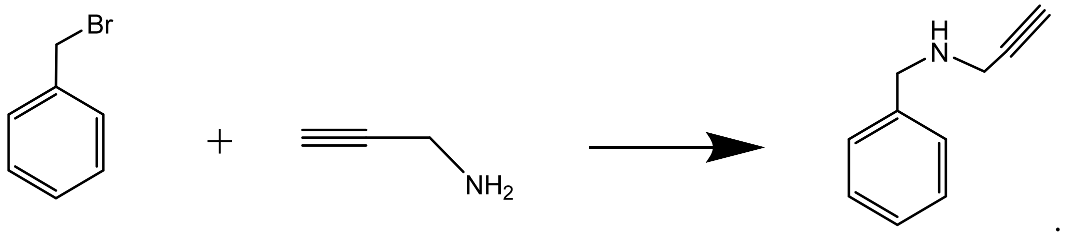

Synthesized according to the previously reported procedure [6]. A reaction mixture of (bromomethyl)benzene (2.9 ml, 24.3 mmol), prop-2-yn-l-amine (5.0 mL, 72.5 mmol) and K_2CO_3 (4.0 g, 29.2 mmol) in anhydrous CH_3CN (70.0 ml) was stirred at 90 °C under N_2 for 12 h. After filtration, the solvent was removed under reduced pressure. The residue was purified by flash chromatography on silica gel (hexane:EtOAc *v*:*v* 10:1) to give pale yellow oil (6.50 g, 94% yield). ^{1}H NMR (400 MHz, $CDCl_3$): δ 7.43-7.20 (m, 5H), 3.91 (s, 2H), 3.46 (d, *J* = 2.4 Hz, 2H), 2.29 (t, *J* = 2.4 Hz, 1H), 1.57 (s, 1H).

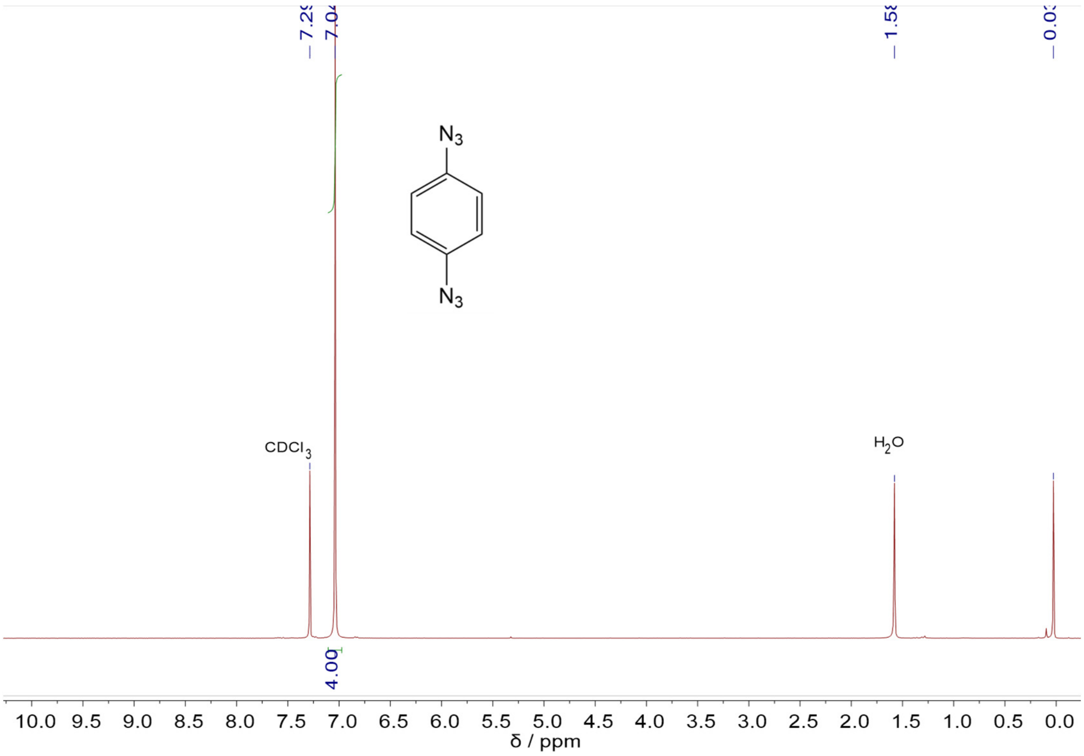

Figure S1. ^{1}H NMR spectrum of 1,4-diazidobenzene in $CDCl_3$ at 298 K.

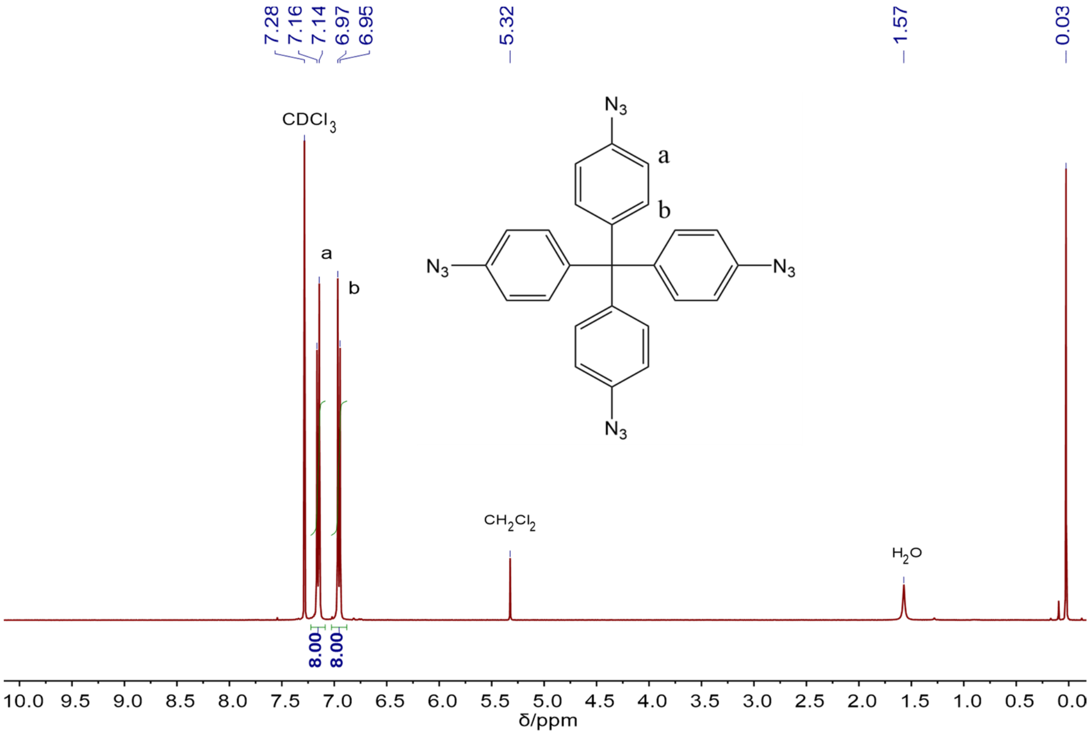

Figure S2. ^{1}H NMR spectrum of tetrakis(4-azidophenyl)methane in $CDCl_3$ at 298 K.

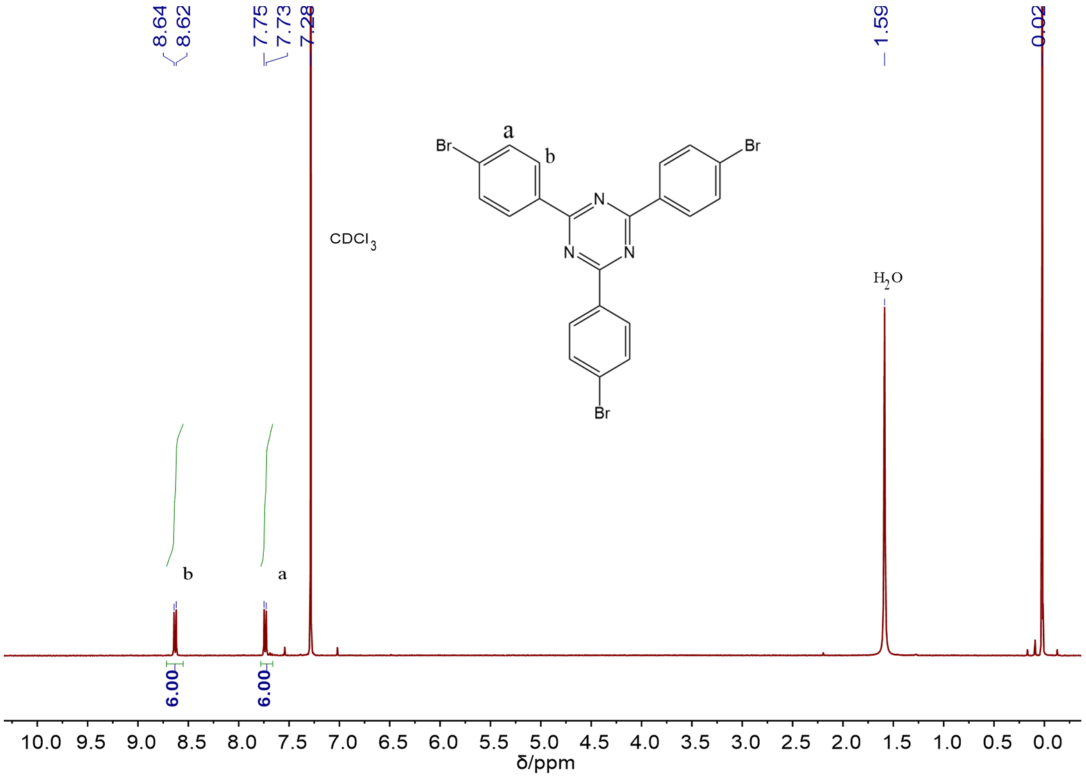

Figure S3. ^{1}H NMR spectrum of 2,4,6-tris(4-bromophenyl)-1,3,5-triazine in $CDCl_3$ at 298 K.

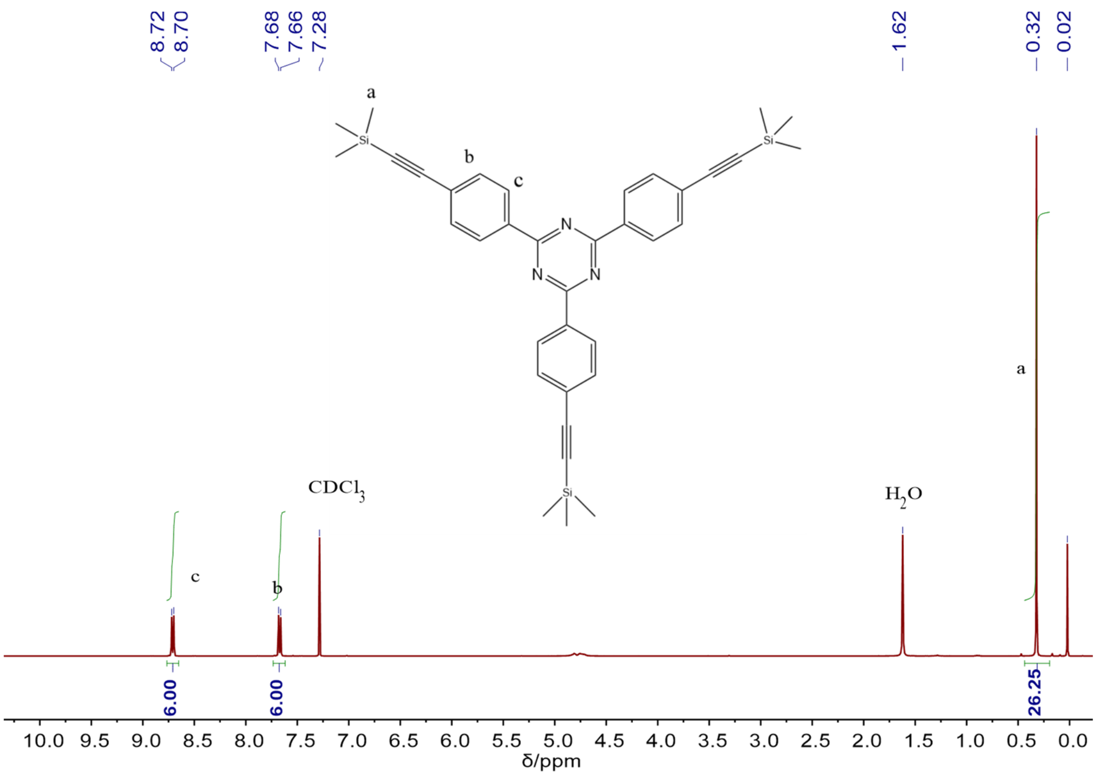

Figure S4. ^{1}H NMR spectrum of 2,4,6-tris(4-((trimethylsilyl)ethynyl)-phenyl)-1,3,5-triazine in $CDCl_3$ at 298 K.

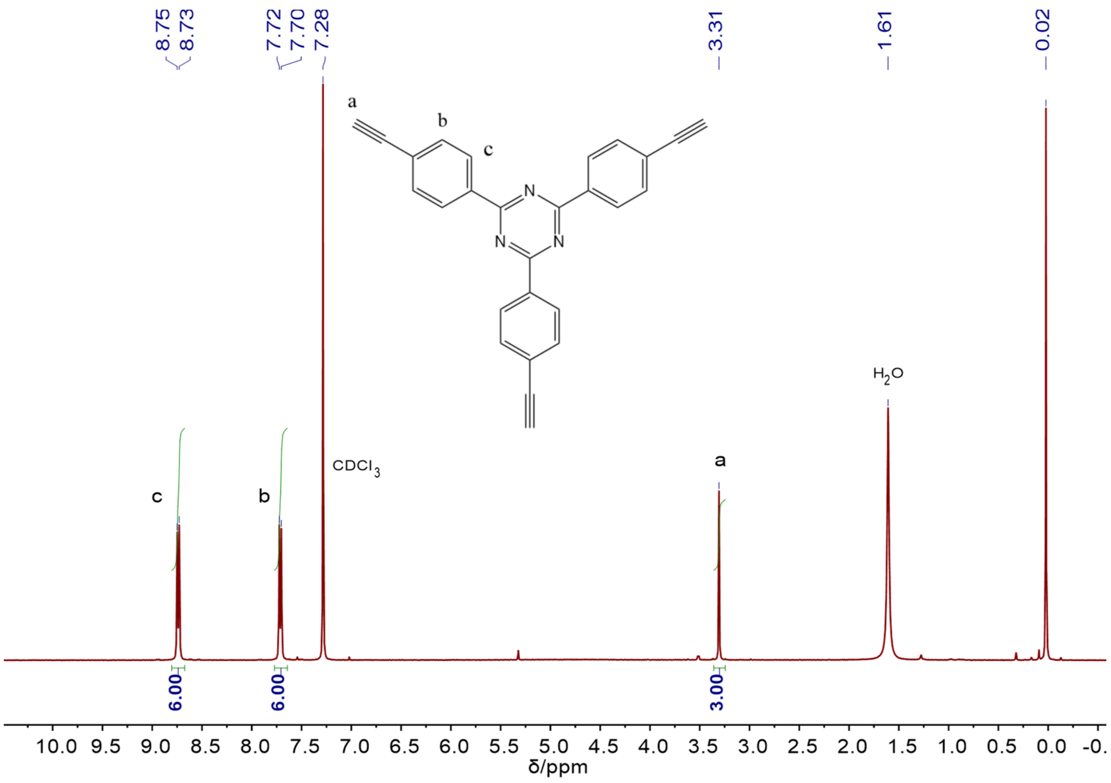

Figure S5. ^{1}H NMR spectrum of 2,4,6-tris(4-ethynylphenyl)-1,3,5-triazine in $CDCl_3$ at 298 K.

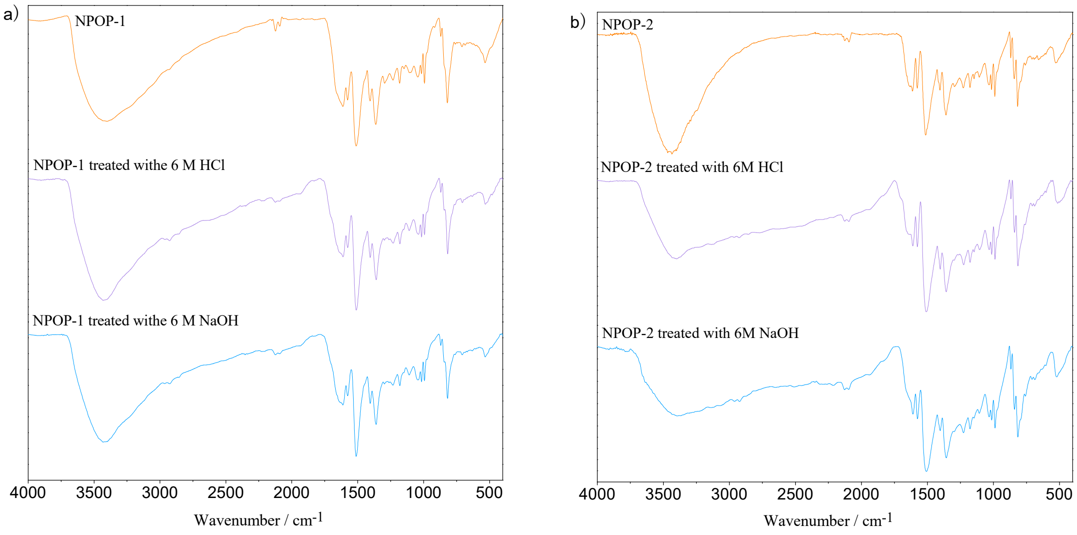

Figure S6. FT-IR spectra of (a) NPOP-1 and (b) NPOP-2 treated with 6 mol L^{-1} HCl (aq.) and 6 mol L^{-1} NaOH (aq.).

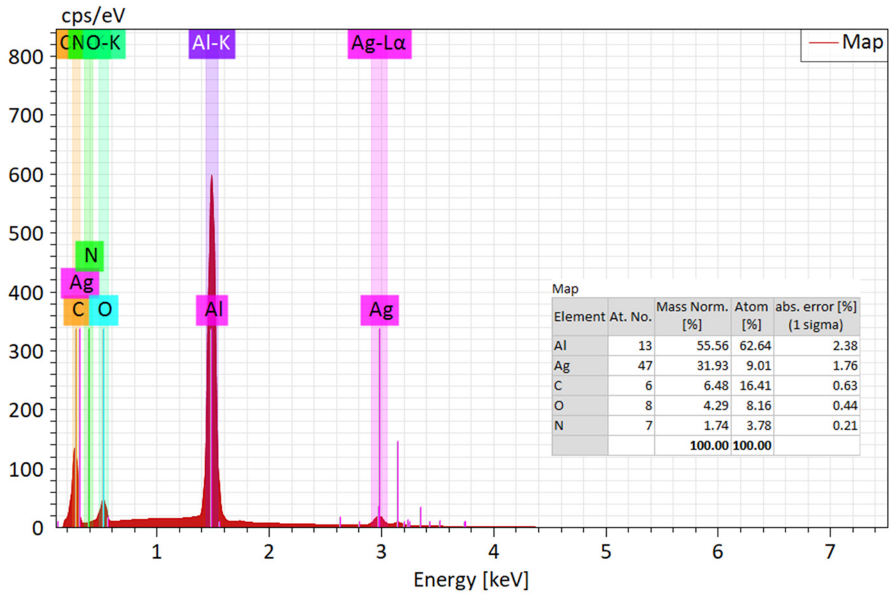

Figure S7. EDS spectrum of Ag@NPOP-1.

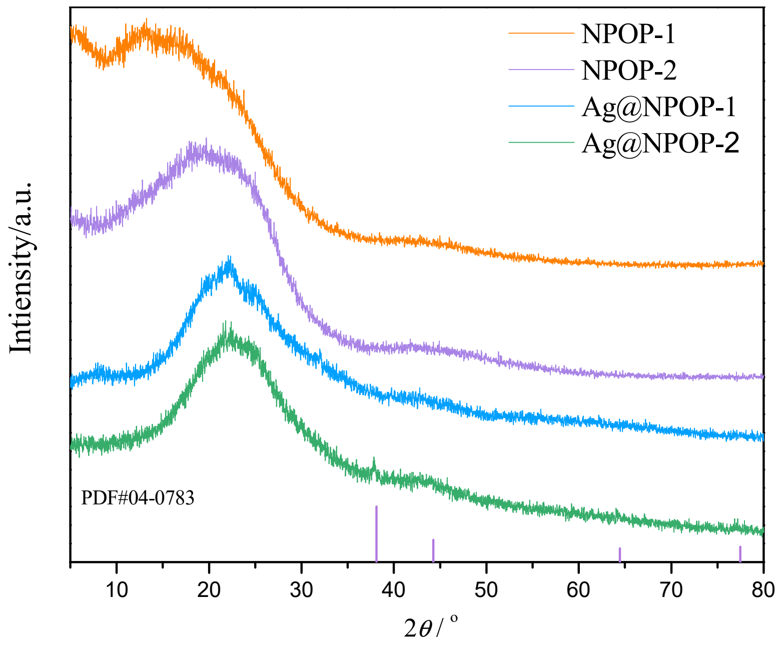

Figure S8. PXRD patterns of NPOP-1, NPOP-2, Ag@NPOP-1 and Ag@NPOP-2.

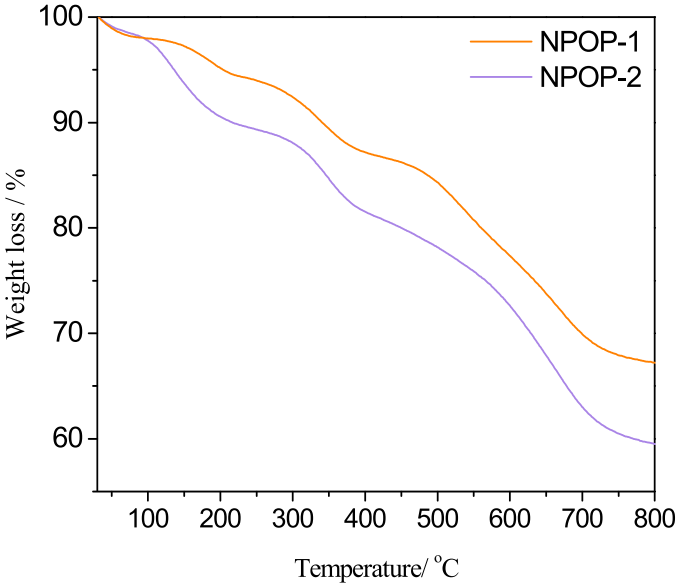

Figure S9. Thermogravimetric profiles of NPOP-1 and NPOP-2.

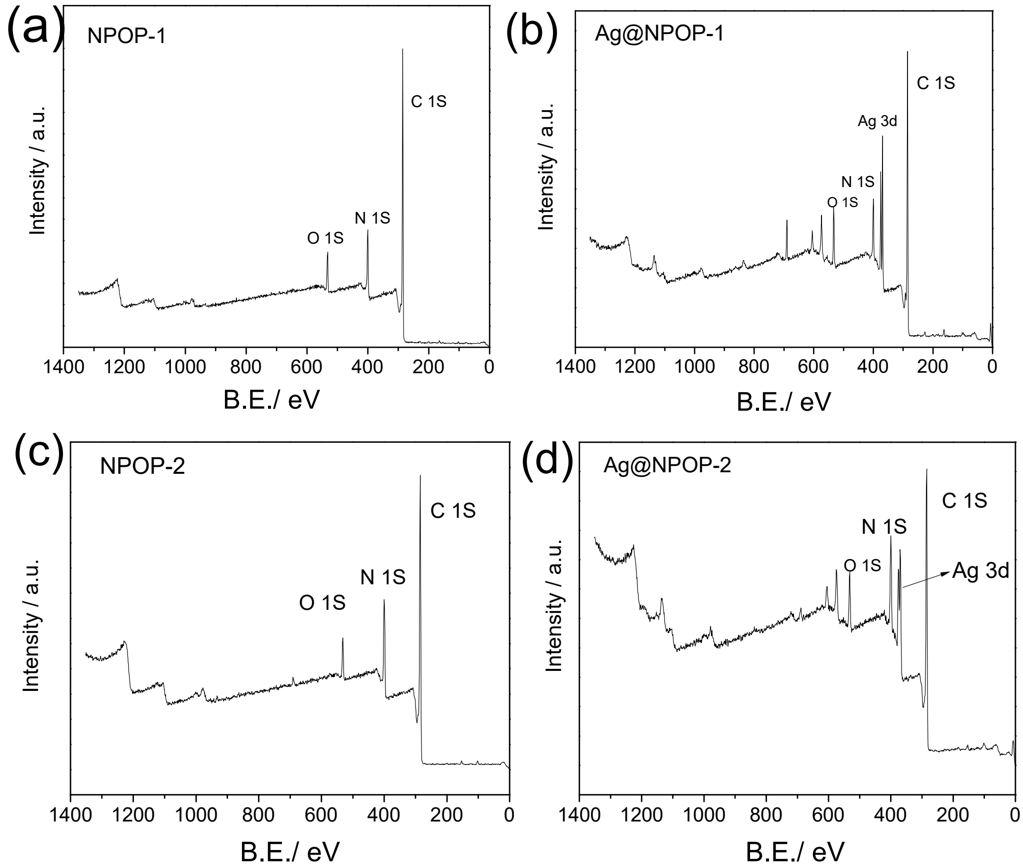

Figure S10. XPS survey spectra of (a) NPOP-1, (b) NPOP-2, (c) Ag@NPOP-1 and (d) Ag@NPOP-2.

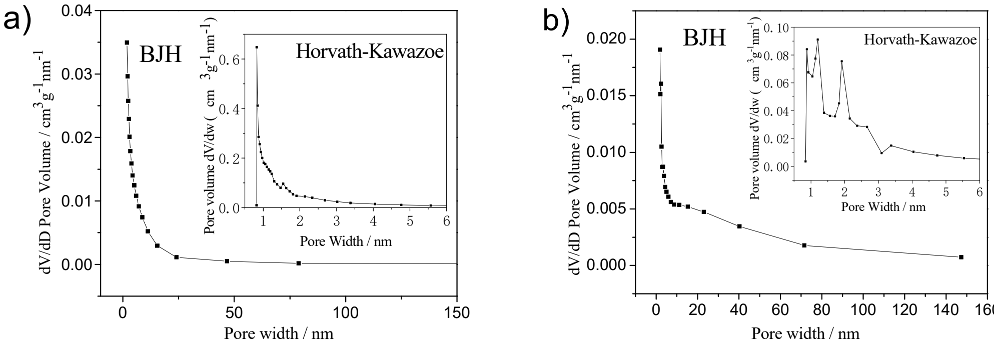

Figure S11. Pore size distributions of (a) NPOP-1 and (b) NPOP-2 calculated by BJH and Horvath-Kawazoe methods.

Method for calculations of isosteric heat (Q_{st}) of CO_2 adsorption

The virial-type equation was first used to fit the CO_2 adsorption isotherm data:

$$\ln P = \ln N + \frac{1}{T}\sum_{i=0}^{m} a_i N^i + \sum_{i=0}^{n} b_i N^i$$

Q_{st} can be calculated by applying the following formula:

$$Q_{st} = -R \sum_{i=0}^{m} a_i N^i$$

N: CO_2 uptake amount (mg g^{-1})
P: Pressure (mmHg)
T: Absolute temperature (K)
a_i, b_i: Empirical constant
R: Universal gas constant = 8.314 J mol^{-1} K^{-1}
Q_{st}: Isosteric heat

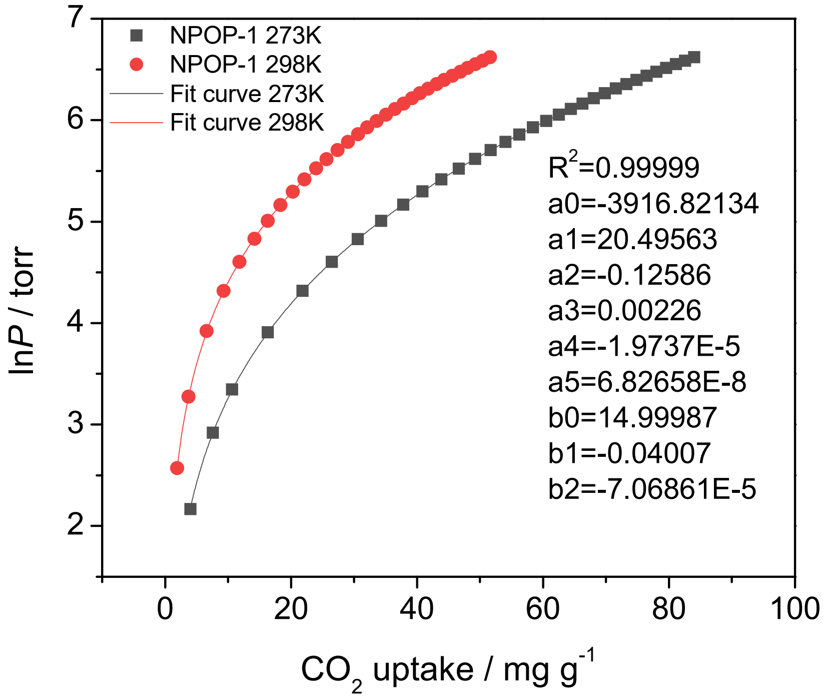

Figure S12. CO_2 isotherms at 273 and 298 K and the virial equation fits of NPOP-1.

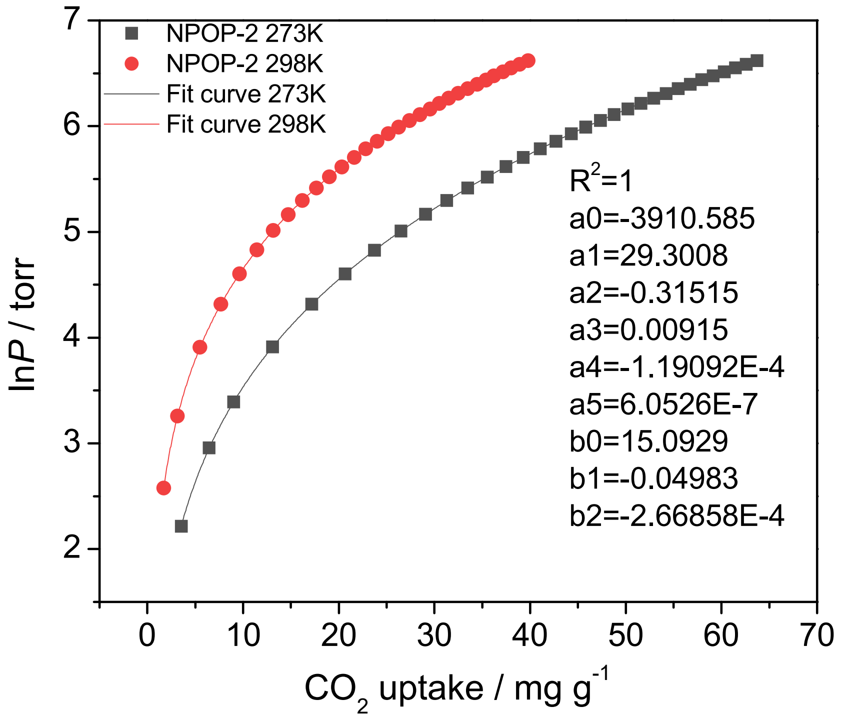

Figure S13. CO_2 isotherms at 273 and 298 K and the virial equation fits of NPOP-2.

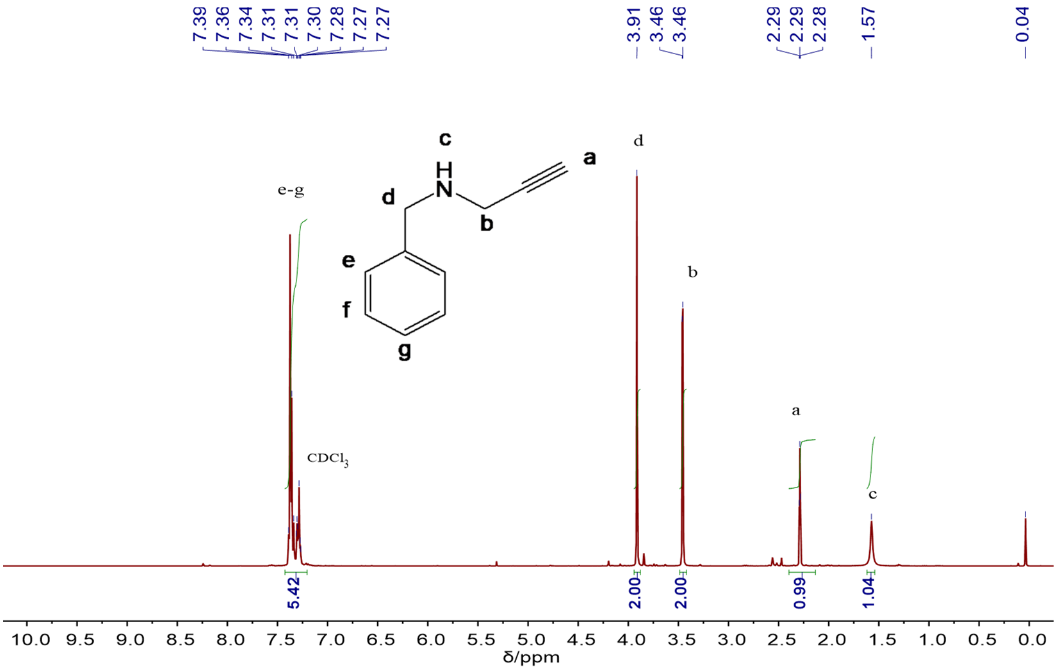

Figure S14. ^{1}H NMR spectrum of *N*-propargylbenzylamine in $CDCl_3$ at 298 K.

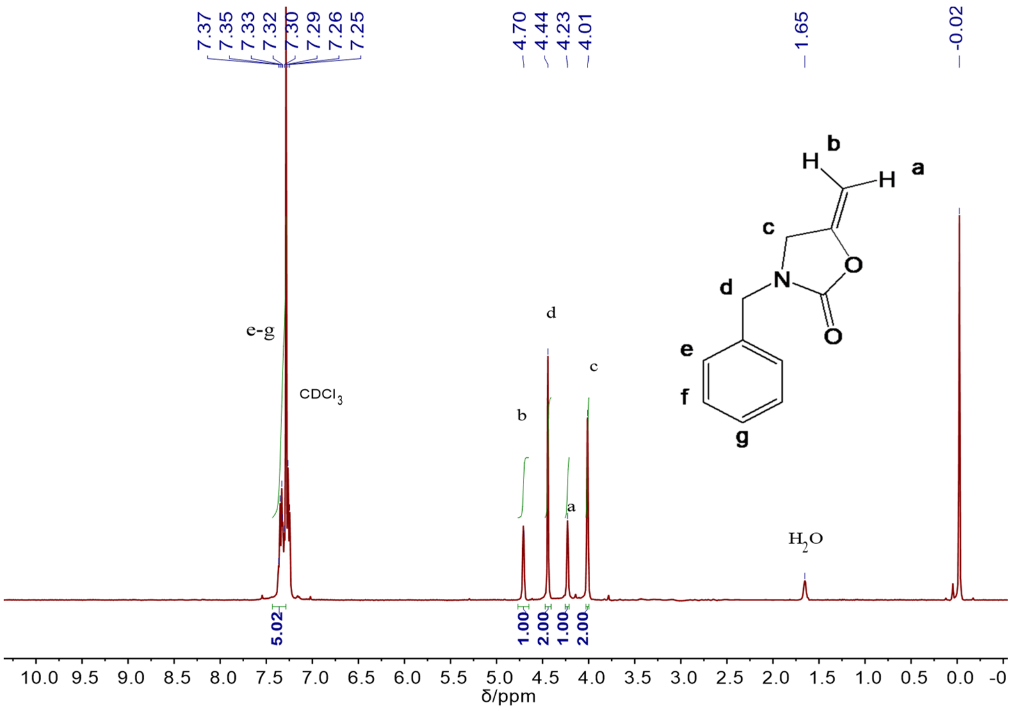

Figure S15. ^{1}H NMR spectrum of 3-benzyl-5-methyleneoxazolidin-2-one in $CDCl_3$ at 298 K.

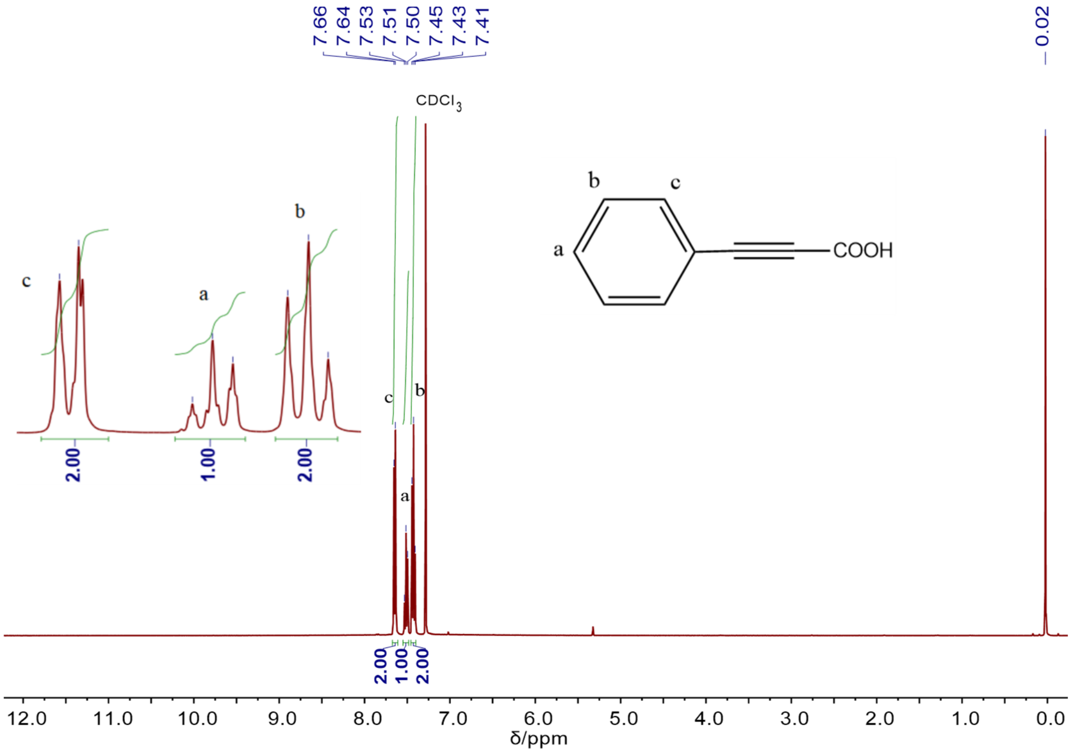

Figure S16. ^{1}H NMR spectrum of phenylpropyolic acid in $CDCl_3$ at 298 K.

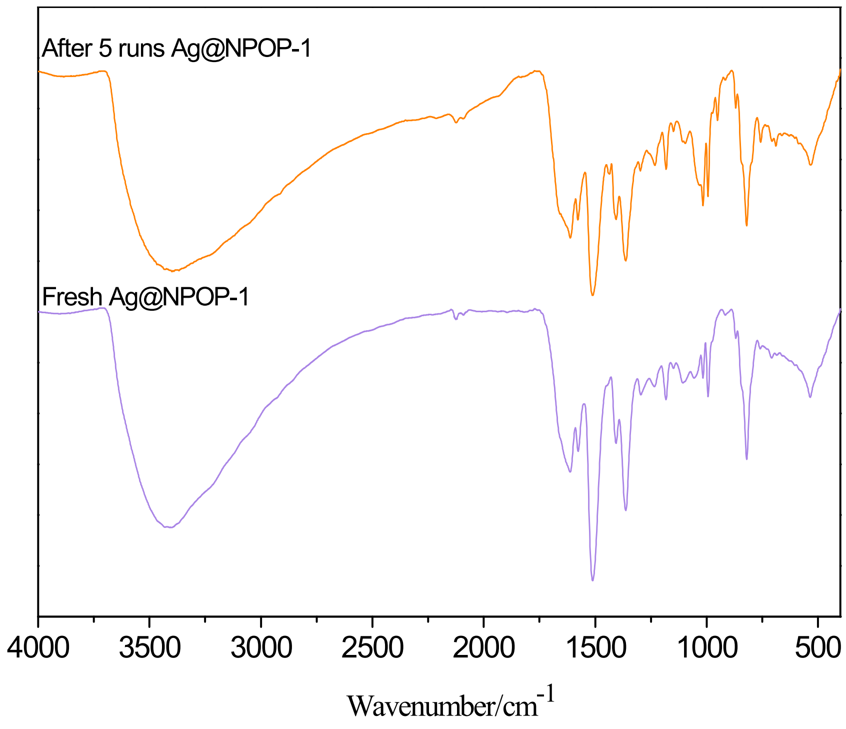

Figure S17. FT-IR spectra of fresh Ag@NPOP-1 and Ag@NPOP-1 after reuse for five runs.

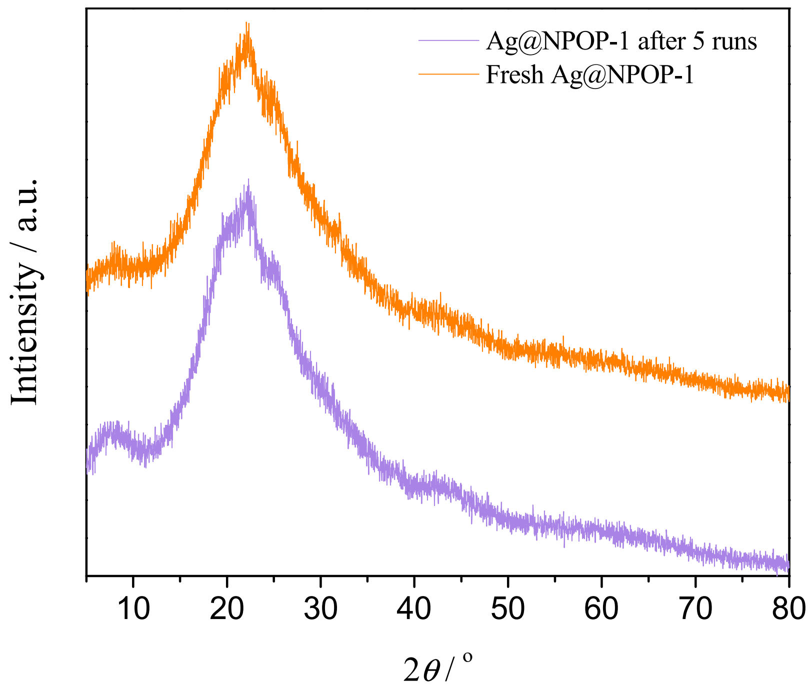

Figure S18. PXRD patterns of fresh Ag@NPOP-1 and Ag@NPOP-1 after reuse for five runs.

Table S1. Elemental analysis of NPOP-1 and NPOP-2.

NPOP	C/%	H/%	N/%	C/N Ratio	C/H Ratio
NPOP-1	66.72	4.56	15.33	4.35	14.63
NPOP-2	63.39	4.05	22.31	2.84	15.65

Table S2. Porosity data of NPOP-1 and NPOP-2.

NPOP	S_{BET}[a]/m^2 g^{-1}	V_{tot}[b]/cm^3 g^{-1}	V_{micro}[c]/cm^3 g^{-1}	V_{micro}/V_{tot}

NPOP-1	481	0.39	0.23	0.59
NPOP-2	233	0.55	0.11	0.20

[a] S_{BET} is the BET specific surface area.

[b] V_{tot} is the total pore volume measured by using the adsorption branch of N_2 isotherm at P/P_0 = 0.99.

[c] V_{micro} is the pore volume measured by N_2 adsorption isotherm using Horvath-Kawazoe method.

Table S3. Comparison of CO_2 absorption capability with previous reported porous materials.

**Material**	**S_{BET}/ m^2 g^{-1}**	**CO_2 uptake / mg g^{-1} at 273 K, 1 atm**	**CO_2 sorption capacity in per unit BET surface area/mg m^{-2}**	**Q_{st} for CO_2[a] /kJ mol^{-1}**	**Ref.**
BCM-CTF-4	232	68	0.29	34.0	[7]
CTF-CSU40	326	69	0.21	38.5	[8]
CTF-CSU36@post	625	87	0.14	34.4	[8]
TPA-TCIF(BD)	2907	77.3	0.027	33.7	[10]
PIN1	458	79.2	0.173	31.0	[11]
IT-POP-1	245	75.0	0.31	32.9	[12]
PSN-TAPA	419	74	0.18		[13]
TPT–COF–5	1747	59.4	0.034	30.6	[14]
TFM-1	738	76.1	0.1	27.0	[15]
T-PIL	128.9	16.0	0.12	29.9	[16]
CTF-Cl-1	516	78.6	0.15	37.5	[17]
PCBZ	341	49.7	0.15	38.6	[18]
CuPor-BPDC	442	55.0	0.124		[19]
ILCOF-1	2723	60.0	0.022		[20]
TCMP-5	494	53.68	0.11		[21]
CTFs	667.7	61.9	0.093		[22]
POP-Byp	1123	70	0.062	25.6	[23]
TFMT-550	421	106.5	0.25	29.8	[24]
KAPS-Py	199	47.7	0.24		[25]
NPOP-1	481	84.0	0.17	32.6	This work
NPOP-2	223	63.7	0.27	32.5	This Work

[a] Value at zero coverage.

Table S4. Comparison with previous reported catalysts for carboxylative cyclization of propargylic amines with CO_2.

**Catalyst**	**Time /h**	**T/°C**	**Solvent**	**Base**	**Yield /%**	**TON**	**TOF/h^{-1}**	**Ref.**
AgN@COF (20)	10	55	CH_3CN	DBU	94.0	120	12	[26]
Cu-NPs@COF	12	60	H_2O:EtOH	DMAB	95.0	25.6	2.1	[27]
Pd@BBA-2	10	60	DMSO	/	98.0	89.1	8.9	[28]
CuOAc+(PhSe)$_2$	11	R.T.	DMSO	DTBP/ Cs_2CO_3	90.0	4.5	0.41	[29]
$((nC_4H_9)_4N)_6(\alpha\text{-}SiW_{11}O_{39}Cu)$	20	60	EtOH	/	95	38	1.9	[30]
Ag-rGO-3	12	30	/	(N_{4444})(Triz)	90	13.4	1.1	[31]
$CoBr_2$	21	60	THF	TBD	99	10	0.48	[32]
Ag@TpPa-1	18	60	CH_3CN	DBU	96	312	17	[33]
$Zn(OAc)_2$	12	60	CH_3CN	TBD	99	10	0.83	[34]
Pd@SCS	0.16	80	DMSO	/	83	83	518	[35]
TOS-Ag4	24	25	CH_3CN	DBU	99	100	4.1	[36]
Ag@2,6-FPP-TAPT	2	50	CH_3CN	DBU	99	1928	964	[37]
Ag@NPOP-1	2	50	CH_3CN	DBU	97.0	2250	1125.1	This work

Ag@NPOP-2	2	50	CH_3CN	DBU	93.0	1555.2	777.6	This work
Ag@NPOP-1	2	40	CH_3CN	DBU	86.0	1995	997.5	This work
Ag@NPOP-1	2	30	CH_3CN	DBU	40.0	928	464.0	This work

Table S5. Comparison with previous reported catalysts for 3-phenylpropiolic acid from CO_2 and 1-ethynylbenzene.

Catalyst	Time /h	*T*/°C	Solvent	Base	Yield /%	TON	TOF/h^{-1}	Ref.
Ag-HMP-2	12	80	DMF	Cs_2CO_3	98.0	82.3	6.9	[38]
Ag@PHNCT	20	50	DMSO	Cs_2CO_3	98.0	93.6	4.7	[39]
0.10Ag/IRFC	20	70	DMSO	Cs_2CO_3	99.0	173.0	8.6	[40]
Ag@NOMP	12	50	DMSO	Cs_2CO_3	96.0	891.8	74.3	[41]
Ag@p-CTF-250	16	50	DMSO	Cs_2CO_3	95.0	23.8	1.5	[22]
1.5Ag@SiO2	20	70	DMF	Cs_2CO_3	95.7	16.7	0.8	[42]
0.5Ag@ZIF-8	20	40	DMF	Cs_2CO_3	97.0	87.0	4.3	[43]
TpBpy-Cu-14	24	60	DMSO	Cs_2CO_3	95.0	75.3	3.1	[44]
Ag NPs/MCC	16	50	DMSO	Cs_2CO_3	99.0	2224.8	139.0	[45]
Ag@MIL-101-1C	15	50	DMF	Cs_2CO_3	96.5	35.7	2.4	[46]
NHCAuCl-COF	16	50	DMSO	Cs_2CO_3	96.0	192.0	12.0	[47]
Ag@NPOP-1	12	60	DMSO	Cs_2CO_3	94.0	1090.3	90.9	This Work
Ag@NPOP-2	12	60	DMSO	Cs_2CO_3	92.1	770.1	64.2	This Work

References

1. Siddiki, A.A.; Takale, B.S.; Telvekar, V.N. One Pot Synthesis of Aromatic Azide Using Sodium Nitrite and Hydrazine Hydrate. *Tetrahedron Lett.* **2013**, *54*, 1294–1297.
2. Yoon, J.; Choi, H.M.; Lee, S.J. Cu(II)Cl_2 Containing Bispyridine-Based Porous Organic Polymer Support Prepared via Alkyne–azide Cycloaddition as a Heterogeneous Catalyst for Oxidation of Various Olefins. *New, J. Chem.* **2020**, *44*, 9149–9152.
3. Liu, Z.; Su, Q.; Ju, P.; Li, X.; Li, G. Wu, Q.; Yang, B. A Hydrophilic Covalent Organic Framework for Photocatalytic Oxidation of Benzylamine in Water. *Chem. Commun.* **2020**, *56*, 766–769.
4. Qiang, H.; Chen, T.; Wang, Z.; Li, W.; Guo, Y.; Yang, J.; Jia, X.; Yang, H.; Hu, W.; Wen, K. Pillar[5]Arene Based Conjugated Macrocycle Polymers with Unique Photocatalytic Selectivity. *Chin. Chem. Lett.* **2020**, *31*, 3225–3229.
5. Klinkebiel, A.; Beyer, O.; Lüning, U. Substituted 1,3,5-Triazine Hexacarboxylates as Potential Linkers for MOFs. *Molecules* **2019**, *24*, 3480.
6. Singudas, R.; Reddy, N.C.; Rai, V. Sensitivity Booster for Mass Detection Enables Unambiguous Analysis of Peptides, Proteins, Antibodies, and Protein Bioconjugates. *Chem. Commun.* **2019**, *55*, 9979–9982.
7. Dong, B.; Wang, D.-Y.; Wang, W.-J. Post-Functionalization of Hydroxyl-Appended Covalent Triazine Framework via Borrowing Hydrogen Strategy for Effective CO_2 Capture. *Micropor. Mesopor. Mater.* **2020**, *292*, 109765.
8. Fu, Y.; Wang, Z.; Li, S.; He, X.; Pan, C.; Yan, J. Yu, G. Functionalized Covalent Triazine Frameworks for Effective CO_2 and SO_2 Removal. *ACS Appl. Mater. Interfaces* **2018**, *10*, 36002–36009.
9. Fu, Y.; Wang, Z.; Fu, X.; Yan, J.; Liu, C.; Pan, C.; Yu, G. Acid/Hydrazide-Appended Covalent Triazine Frameworks for Low-pressure CO_2 Capture: Pre-designable or Post-synthesis Modification. *J. Mater. Chem. A* **2017**, *5*, 21266–21274.
10. Puthiaraj, P.; Kim, H.S.; Yu, K.; Ahn, W.-S. Triphenylamine-Based Covalent Imine Framework for CO_2 Capture and Catalytic Conversion into Cyclic Carbonates. *Microporous Mesoporous Mater.* **2020**, *297*, 110011.
11. Popp, N.; Homburg, T.; Stock, N.; Senker, J. Porous Imine-Based Networks with Protonated Imine Linkages for Carbon Dioxide Separation from Mixtures with Nitrogen and Methane. *J. Mater. Chem. A* **2015**, *3*, 18492–18504.
12. Zhong, H.; Su, Y.; Chen, X.; Li, X.; Wang, R. Imidazolium- and Triazine-Based Porous Organic Polymers for Heterogeneous Catalytic Conversion of CO_2 into Cyclic Carbonates. *ChemSusChem* **2017**, *10*, 4855–4863.
13. Li, G.; Zhang, B.; Yan, J.; Wang, Z. Micro- and Mesoporous Poly(Schiff-base)s Constructed from Different Building Blocks and Their Adsorption Behaviors Towards Organic Vapors and CO_2 Gas. *J. Mater. Chem. A* **2014**, *2*, 18881–18888.
14. El-Mahdy, A.F.M.; Kuo, C.-H.; Alshehri, A.; Young, C.; Yamauchi, Y.; Kim, J.; Kuo, S.-W. Strategic Design of Triphenylamine- and Triphenyltriazine-based Two-dimensional Covalent Organic Frameworks for CO_2 Uptake and Energy Storage. *J. Mater. Chem. A* **2018**, *6*, 19532–19541.
15. Xu, Y.; Jin, S.; Xu, H.; Nagai, A.; Jiang, D. Conjugated Microporous Polymers: Design, Synthesis and Application. *Chem. Soc. Rev.* **2013**, *42*, 8012–8031.
16. Fang, X.; Liu, C.; Yang, L.; Yu, T.; Zhai, D.; Zhao, W.; Deng, W.-q. Bifunctional poly(ionic liquid) Catalyst with Dual-active-center for CO_2 Conversion: Synergistic Effect of Triazine and Imidazolium Motifs. *J. CO_2 Util.* **2021**, *54*, 101778.
17. Xu, G.; Zhu, Y.; Xie, W.; Zhang, S.; Yao, C.; Xu, Y. Porous Cationic Covalent Triazine-Based Frameworks as Platforms for Efficient CO_2 and Iodine Capture. *Chem.-Asian, J.* **2019**, *14*, 3259–3263.

18. Saleh, M.; Baek, S.B.; Lee, H.M.; Kim, K.S. Triazine-Based Microporous Polymers for Selective Adsorption of CO_2. *J. Phys. Chem. C* **2015**, *119*, 5395–5402.
19. Neti, V.S.P.K.; Wu, X.; Deng, S.; Echegoyen, L. Selective CO_2 Capture in An Imine Linked Porphyrin Porous Polymer. *Polym. Chem.* **2013**, *4*, 4566–4569.
20. Rabbani, M.G.; Sekizkardes, A.K.; Kahveci, Z.; Reich, T.E.; Ding, R.; El-Kaderi, H.M. A 2D Mesoporous Imine-Linked Covalent Organic Framework for High Pressure Gas Storage Applications. *Chem. - Eur. J.* **2013**, *19*, 3324–3328.
21. Ren, S.; Dawson, R.; Laybourn, A.; Jiang, J.-x.; Khimyak, Y.; Adams, D.J.; Cooper, A.I. Functional Conjugated Microporous Polymers: From 1,3,5-benzene to 1,3,5-triazine. *Polym. Chem.* **2012**, *3*, 928–934.
22. Liu, J.; Zhang, X.; Wen, B.; Li, Y.; Wu, J.; Wang, Z.; Wu, T.; Zhao, R.; Yang, S. Pre-carbonized Nitrogen-rich Polytriazines for The Controlled Growth of Silver nanoparticles: Catalysts for Enhanced CO_2 Chemical Conversion at Atmospheric Pressure. *Catal. Sci. Technol.* **2021**, *11*, 3119–3127.
23. Dai, Z.; Sun, Q.; Liu, X.; Guo, L.; Li, J.; Pan, S.; Bian, C.; Wang, L.; Hu, X.; Meng, X.; Zhao, L.; Deng, F.; Xiao, F.-S. A Hierarchical Bipyridine-Constructed Framework for Highly Efficient Carbon Dioxide Capture and Catalytic Conversion. *ChemSusChem* **2017**, *10*, 1186–1192.
24. Zhu, X.; Chai, S.; Tian, C.; Fulvio, P.F.; Han, K.S.; Hagaman, E.W.; Veith, G.M.; Mahurin, S.M.; Brown, S.; Liu, H.; Dai, S. Synthesis of Porous, Nitrogen-Doped Adsorption/Diffusion Carbonaceous Membranes for Efficient CO_2 Separation. *Macromol. Rapid Commun.* **2013**, *34*, 452–459.
25. Wu, Z.; Liu, Q.; Yang, X.; Ye, X.; Duan, H.; Zhang, J.; Zhao, B.; Huang, Y. Knitting Aryl Network Polymers-Incorporated Ag Nanoparticles: A Mild and Efficient Catalyst for the Fixation of CO_2 as Carboxylic Acid. *ACS Sustainable Chem. Eng* **2017**, *5*, 9634–9639.
26. Islam, S.S.; Biswas, S.; Ali Molla, R.; Yasmin, N.; Islam, S.M. Green Synthesized AgNPs Embedded in COF: An Efficient Catalyst for the Synthesis of 2-Oxazolidinones and α-Alkylidene Cyclic Carbonates via CO_2 Fixation. *ChemNanoMat* **2020**, *6*, 1386–1397.
27. Khatun, R.; Biswas, S.; Biswas, I.H.; Riyajuddin, S.; Haque, N.; Ghosh, K.; Islam, S.M. Cu-NPs@COF: A Potential Heterogeneous Catalyst for CO_2Fixation to Produce 2-Oxazolidinones as Well as Benzimidazoles under Moderate Reaction Conditions. *J. CO_2 Util.* **2020**, *40*, 101180.
28. Ghosh, S.; Riyajuddin, S.; Sarkar, S.; Ghosh, K.; Islam, S.M. Pd NPs Decorated on POPs as Recyclable Catalysts for the Synthesis of 2-Oxazolidinones from Propargylic Amines via Atmospheric Cyclizative CO_2 Incorporation. *ChemNanoMat* **2020**, *6*, 160–172.
29. Chen, J.-M.; Qi, L.; Zhang, L.; Li, L.-J., Hou, C.-Y.; Li, W.; Wang, L.-J. Copper/DTBP-Promoted Oxyselenation of Propargylic Amines with Diselenides and CO_2: Synthesis of Selenyl 2-Oxazolidinones. *J. Org. Chem.* **2020**, *85*, 10924–10933.
30. Wang, M.-Y.; Song, Q.-W.; Ma, R.; Xie, J.-N.; He, L.-N. Efficient Conversion of Carbon Dioxide at Atmospheric Pressure to 2-Oxazolidinones Promoted by Bifunctional Cu(ii)-Substituted Polyoxometalate-Based Ionic Liquids. *Green Chem.* **2016**, *18*, 282–287.
31. Zhang, X.; Chen, K.-H.; Zhou, Z.-H.; He, L.-N. Reduced Graphene Oxide Supported Ag Nanoparticles: An Efficient Catalyst for CO_2 Conversion at Ambient Conditions. *ChemCatChem* **2020**, *12*, 4825–4830.
32. Zhou, Z.-H.; Xia, S.-M.; Huang, S.-Y.; Huang, Y.-Z.; Chen, K.-H.; He, L.-N. Cobalt-Based Catalysis for Carboxylative Cyclization of Propargylic Amines with CO_2 at Atmospheric Pressure. *J. CO_2 Util.* **2019**, *34*, 404–410.
33. Ghosh, S.; Molla, R.A.; Kayal, U.; Bhaumik, A.; Islam, S.M. Ag NPs Decorated on a COF in the Presence of DBU as an Efficient Catalytic System for the Synthesis of Tetramic Acids via CO_2 Fixation into Propargylic Amines at Atmospheric Pressure. *Dalton Trans.* **2019**, *48*, 4657–4666.
34. Liu, X.; Wang, M.-Y.; Wang, S.-Y.; Wang, Q.; He, L.-N. In Situ Generated Zinc(II) Catalyst for Incorporation of CO_2 into 2-Oxazolidinones with Propargylic Amines at Atmospheric Pressure. *ChemSusChem* **2017**, *10*, 1210–1216.
35. Brunel, P.; Monot, J.; Kefalidis, C.E.; Maron, L.; Martin-Vaca, B.; Bourissou, D. Valorization of CO_2: Preparation of 2-Oxazolidinones by Metal–Ligand Cooperative Catalysis with SCS Indenediide Pd Complexes. *ACS Catal.* **2017**, *7*, 2652–2660.
36. Chang, Z.; Jing, X.; He, C.; Liu, X.; Duan, C. Silver Clusters as Robust Nodes and π–Activation Sites for the Construction of Heterogeneous Catalysts for the Cycloaddition of Propargylamines. *ACS Catal.* **2018**, *8*, 1384–1391.
37. Zhang, Y.; Lan, X.; Yan, F.; He, X.; Wang, J., Ricardez-Sandoval, L.; Chen, L.; Bai, G. Controllable Encapsulation of Silver Nanoparticles by Porous Pyridine-Based Covalent Organic Frameworks for Efficient CO_2 Conversion Using Propargylic Amines. *Green Chem.* **2022**, *24*, 930–940.
38. Ghosh, S.; Ghosh, A.; Riyajuddin, S.; Sarkar, S.; Chowdhury, A.H.; Ghosh, K.; Islam, S.M. Silver Nanoparticles Architectured HMP as a Recyclable Catalyst for Tetramic Acid and Propiolic Acid Synthesis through CO_2 Capture at Atmospheric Pressure. *ChemCatChem* **2020**, *12*, 1055–1067.
39. Lan, X.; Li, Q.; Cao, L.; Du, C.; Ricardez-Sandoval, L.; Bai, G. Rebuilding Supramolecular Aggregates to Porous Hollow N-Doped Carbon Tube Inlaid with Ultrasmall Ag Nanoparticles: A Highly Efficient Catalyst for CO_2Conversion. *Appl. Surf. Sci.* **2020**, *508*, 145220.
40. Zhang, Z.; Shi, J.; Zhu, T.; Zhang, L.; Wei, W. Nitrogen-Doped Mesoporous Carbon Single Crystal-Based Ag Nanoparticles for Boosting Mild CO_2 Conversion with Terminal Alkynes. *J. Colloid Interface Sci.* **2022**, *627*, 81–89.
41. Zhang, W.; Mei, Y.; Huang, X.; Wu, P.; Wu, H.; He, M. Size-Controlled Growth of Silver Nanoparticles onto Functionalized Ordered Mesoporous Polymers for Efficient CO_2 Upgrading. *ACS Appl. Mater. Interfaces* **2019**, *11*, 44241–44248.
42. Li, M.; Zhang, L.; Zhang, Z.; Shi, J.; Liu, Y.; Chen, J.; Sun, N.; Wei, W. SiO_2-Coated Ag Nanoparticles for Conversion of Terminal Alkynes to Propolic Acids via CO_2 Insertion. *ACS Appl. Nano Mater.* **2021**, *4*, 7107–7115.

43. Shi, J.; Zhang, L.; Sun, N.; Hu, D.; Shen, Q.; Mao, F.; Gao, Q.; Wei, W. Facile and Rapid Preparation of Ag@ZIF-8 for Carboxylation of Terminal Alkynes with CO_2 in Mild Conditions. *ACS Appl. Mater. Interfaces* **2019**, *11*, 28858–28867.
44. Bu, R.; Zhang, L.; Gao, L.-L.; Sun, W.-J.; Yang, S.-L.; Gao, E.-Q. Copper(I)-Modified Covalent Organic Framework for CO_2 Insertion to Terminal Alkynes. *Mol. Catal.* **2021**, *499*, 111319.
45. Shah, D.J.; Sharma, A.S.; Shah, A.P.; Sharma, V.S.; Athar, M.; Soni, J.Y. Fixation of CO_2 as a Carboxylic Acid Precursor by Microcrystalline Cellulose (MCC) Supported Ag NPs: A More Efficient, Sustainable, Biodegradable and Eco-Friendly Catalyst. *New, J. Chem.* **2019**, *43*, 8669–8676.
46. Liu, X.-H.; Ma, J.-G.; Niu, Z.; Yang, G.-M.; Cheng, P. An Efficient Nanoscale Heterogeneous Catalyst for the Capture and Conversion of Carbon Dioxide at Ambient Pressure. *Angew. Chem. Int. Ed.* **2015**, *54*, 988–991.
47. Li, Y.; Dong, Y.; Kan, J.-L.; Wu, X.; Dong, Y.-B. Synthesis and Catalytic Properties of Metal–N-Heterocyclic-Carbene-Decorated Covalent Organic Framework. *Org. Lett.* **2020**, *22*, 7363–7368.
